# Integrating Clinical Phenotype With Multiomics Analyses of Human Cardiac Tissue Unveils Divergent Metabolic Remodeling in Genotype-Positive and Genotype-Negative Patients With Hypertrophic Cardiomyopathy

**DOI:** 10.1161/CIRCGEN.123.004369

**Published:** 2024-06-10

**Authors:** Edgar E. Nollet, Maike Schuldt, Vasco Sequeira, Aleksandra Binek, Thang V. Pham, Stephan A.C. Schoonvelde, Mark Jansen, Bauke V. Schomakers, Michel van Weeghel, Fred M. Vaz, Riekelt H. Houtkooper, Jennifer E. Van Eyk, Connie R. Jimenez, Michelle Michels, Kenneth C. Bedi, Kenneth B. Margulies, Cristobal G. dos Remedios, Diederik W.D. Kuster, Jolanda van der Velden

**Affiliations:** Department of Physiology (E.E.N., M.S., D.W.D.K., J.v.d.V.), Amsterdam UMC, the Netherlands.; Department of Medical Oncology, VUmc Cancer Center Amsterdam, OncoProteomics Laboratory (T.V.P., C.R.J.), Amsterdam UMC, the Netherlands.; Laboratory Genetic Metabolic Diseases (B.V.S., M.v.W., F.M.V., R.H.H.), Amsterdam UMC, the Netherlands.; Core Facility Metabolomics (B.V.S., M.v.W., F.M.V.), Amsterdam UMC, the Netherlands.; Emma Center for Personalized Medicine (R.H.H.), Amsterdam UMC, the Netherlands.; Amsterdam Cardiovascular Sciences, Heart Failure and Arrhythmias, the Netherlands (E.E.N., M.S., D.W.D.K., J.v.d.V.).; Department of Translational Science Universitätsklinikum, Deutsches Zentrum für Herzinsuffizienz, Würzburg, Germany (V.S.).; Advanced Clinical Biosystems Research Institute (A.B., J.E.V.E.), Cedars-Sinai Medical Center, Los Angeles, CA.; Department of Biomedical Sciences, Smidt Heart Institute (J.E.V.E.), Cedars-Sinai Medical Center, Los Angeles, CA.; Department of Cardiology, Erasmus MC, Rotterdam, the Netherlands (S.A.C.S., M.M.).; Division of Genetics and Department of Cardiology, UMC Utrecht, the Netherlands (M.J.).; Amsterdam Gastroenterology, Endocrinology and Metabolism, the Netherlands (R.H.H.).; Cardiovascular Institute, Perelman School of Medicine, Philadelphia, PA (K.C.B., K.B.M.).; Sydney Heart Bank, Discipline of Anatomy, Bosch Institute, University of Sydney, NSW, Australia (C.G.d.R.).

**Keywords:** cardiomyopathies, genotype, hypertrophy, lipidomics, metabolism, metabolomics

## Abstract

**BACKGROUND:**

Hypertrophic cardiomyopathy (HCM) is caused by sarcomere gene mutations (genotype-positive HCM) in ≈50% of patients and occurs in the absence of mutations (genotype-negative HCM) in the other half of patients. We explored how alterations in the metabolomic and lipidomic landscape are involved in cardiac remodeling in both patient groups.

**METHODS:**

We performed proteomics, metabolomics, and lipidomics on myectomy samples (genotype-positive N=19; genotype-negative N=22; and genotype unknown N=6) from clinically well-phenotyped patients with HCM and on cardiac tissue samples from sex- and age-matched and body mass index–matched nonfailing donors (N=20). These data sets were integrated to comprehensively map changes in lipid-handling and energy metabolism pathways. By linking metabolomic and lipidomic data to variability in clinical data, we explored patient group–specific associations between cardiac and metabolic remodeling.

**RESULTS:**

HCM myectomy samples exhibited (1) increased glucose and glycogen metabolism, (2) downregulation of fatty acid oxidation, and (3) reduced ceramide formation and lipid storage. In genotype-negative patients, septal hypertrophy and diastolic dysfunction correlated with lowering of acylcarnitines, redox metabolites, amino acids, pentose phosphate pathway intermediates, purines, and pyrimidines. In contrast, redox metabolites, amino acids, pentose phosphate pathway intermediates, purines, and pyrimidines were positively associated with septal hypertrophy and diastolic impairment in genotype-positive patients.

**CONCLUSIONS:**

We provide novel insights into both general and genotype-specific metabolic changes in HCM. Distinct metabolic alterations underlie cardiac disease progression in genotype-negative and genotype-positive patients with HCM.

Hypertrophic cardiomyopathy (HCM) is the most common inherited cardiomyopathy.^1^ Clinical hallmarks of HCM include diastolic impairment and asymmetrical left ventricular hypertrophy, often giving rise to left ventricle outflow tract obstruction.^2,3^ Approximately 50% of all patients harbor a pathogenic heterozygous variant (mutation) in genes encoding sarcomere proteins (ie, genotype-positive [G_positive_]). Despite the expansion of the number of genes for diagnostic screening, no causative mutation is identified in the other half of patients.^4–6^ As a result, it remains unknown what drives disease in these genotype-negative (G_negative_) individuals. Nowadays, the majority of newly diagnosed patients with HCM are G_negative_^5^; hence, insight into disease mechanisms in G_negative_ HCM is warranted.

Impairment of cardiac bioenergetics is a well-established feature in all patients with HCM. This is evident from imaging studies showing lower phosphocreatine/adenosine triphosphate ratios and reduced myocardial external efficiency^7,8^ and from proteomic studies in myectomy tissue samples demonstrating widespread downregulation of proteins involved in mitochondrial energy metabolism.^9,10^ Reports from the past few years have aimed to define the metabolomic and lipidomic signatures of HCM to gain insight into, for example, substrate utilization, formation of toxic lipids, and alterations in biosynthetic pathways. While these studies consistently show downregulation in the oxidation of fatty acids, findings on lipid handling and storage, phospholipids, glycolytic intermediates, amino acids, redox metabolites, and purine/pyrimidine metabolism remain inconclusive.^11–13^ Possible explanations for these reported discrepancies may include methodological differences, intercohort dissimilarities (eg, demographics), and suboptimal matching to scarcely available tissue samples from nonfailing donor hearts.

Whereas the underlying cause of the disease is different in G_positive_ and G_negative_ patients, studies leveraging multiomics analyses of myectomy tissue samples uniformly agree on the absence of differences at the group level between these patient populations.^10–14^ However, we recently demonstrated that mitochondrial dysfunction was tightly linked to the degree of septal hypertrophy in G_negative_ patients, while, in G_positive_ patients, mitochondrial function was not associated with hypertrophy.^15^ This challenges the idea that similar metabolic remodeling underlies disease in G_positive_ and G_negative_ patients, and it remains to be determined whether the metabolome and lipidome correlate differently with cardiac phenotype in G_positive_ and G_negative_ patients.

To address these unresolved matters, we here performed proteomics, metabolomics, and lipidomics on a large number of myectomy samples (N=47) from a clinically well-phenotyped cohort of patients with HCM and cardiac tissue samples from sex- and age-matched and body mass index–matched nonfailing donors (N=20). We provide a comprehensive overview of alterations in lipid-handling and energy metabolism pathways, integrating 3 complementary omics analyses. By linking metabolomic and lipidomic profiles to cardiac phenotype, we unveil that pathological cardiac remodeling and fatty acid oxidation impairment in HCM were not associated with increased fatty acid storage or ceramide-induced lipotoxicity. Moreover, in G_negative_ patients, we observed striking negative associations between cardiac remodeling and abundance of acylcarnitines, redox metabolites, amino acids, pentose phosphate pathway (PPP) intermediates, purines, and pyrimidines. In contrast, in G_positive_ patients, cardiac remodeling was positively associated with levels of redox metabolites, amino acids, PPP intermediates, purines, and pyrimidines. Combined, the present study provides novel insights into general and genotype-specific metabolic alterations in relation to cardiac phenotype in HCM and indicates that differential and even opposite metabolic changes underlie cardiac disease in G_positive_ and G_negative_ patients.

## METHODS

An extended methods section and an overview of the study design (Figure S1) are provided in the Supplemental Material.

### Metabolomics and Lipidomics

Metabolomics and lipidomics analyses were performed on snap-frozen cardiac tissue as previously described.^16,17^ A detailed description is provided in the Supplemental Material. Metabolic pathway enrichment analysis was performed using MetaboAnalyst 5.0 (www.MetaboAnalyst.ca).^18^

### Proteomics

Proteomics analyses were performed on cardiac tissue samples that were fractionated according to the IN-Seq method as described previously,^19^ followed by liquid chromatography-tandem mass spectrometry according to a previously published workflow.^20^ A detailed description is provided in the Supplemental Material.

### Respirometry

Mitochondrial respiration data used in this study were previously generated.^15^ Here, we analyzed these data in relation to metabolite and lipid levels in corresponding samples.

### Statistical Analyses

Statistical analyses were performed using GraphPad Prism, v9, software. Metabolomics and lipidomics data were log2 transformed and analyzed by the Welch *t* test. *P* values were corrected for multiple testing via the Benjamini-Hochberg false discovery rate procedure. A corrected *P* value (ie, Q-value) <0.05 was considered significant. Correlations were tested using linear regression and are described by the Pearson R coefficient. Because the aim of this study was to explore associations between metabolites/lipids and clinical phenotype/mitochondrial function, we did not correct significance levels of correlations for multiple testing. Data on glycogen levels were analyzed via the Mann-Whitney *U* test when 2 groups were compared or 1-way ANOVA with the Tukey multiple comparisons test when 3 groups were compared; blood pressure data were analyzed via the Student *t* test or the Welch *t* test. These data are displayed as mean±SEM. Categorical distributions are presented as percentages and were analyzed using the Fisher exact test.

### Study Approval

Study protocols were approved by local medical ethics review committees. Before surgery, written informed consent was obtained from all patients.

### Data Availability Statement

Metabolomics and lipidomics data have been submitted to the MetaboLights database under accession number MTBLS8695.^21^ Proteomics data are available via the ProteomeXchange Consortium via the PRIDE partner repository with the data set identifier PXD046187.^22^ Processed metabolomics, lipidomics, and proteomics data are furthermore available as a Supplemental Material (Multi-Omics Data Sets). The remaining data underlying this article will be shared on reasonable request to the corresponding author.

## RESULTS

We performed multiomic profiling of myectomy tissue from clinically well-phenotyped patients with HCM and cardiac tissue from nonfailing donors to comprehensively map metabolic alterations in HCM. Echocardiographic data were acquired as described previously^23^ and are summarized in Table S1. Detailed individual information of each patient and donor is available in Table S2. Patients with obstructive HCM and nonfailing donors were comparable in terms of sex distribution, age, and body mass index. G_positive_ and G_negative_ patients did not differ demographically and displayed similar indexed septal thickness and diastolic function. Left ventricle outflow tract pressure gradients were significantly higher in G_negative_ versus G_positive_ patients, in line with observations in the NHLBI HCM Registry.^5,24^

### Metabolomics Reveals Impaired Fatty Acid Oxidation and Alterations in Glycolytic and Biosynthetic Pathways in HCM

**Figure 1.**
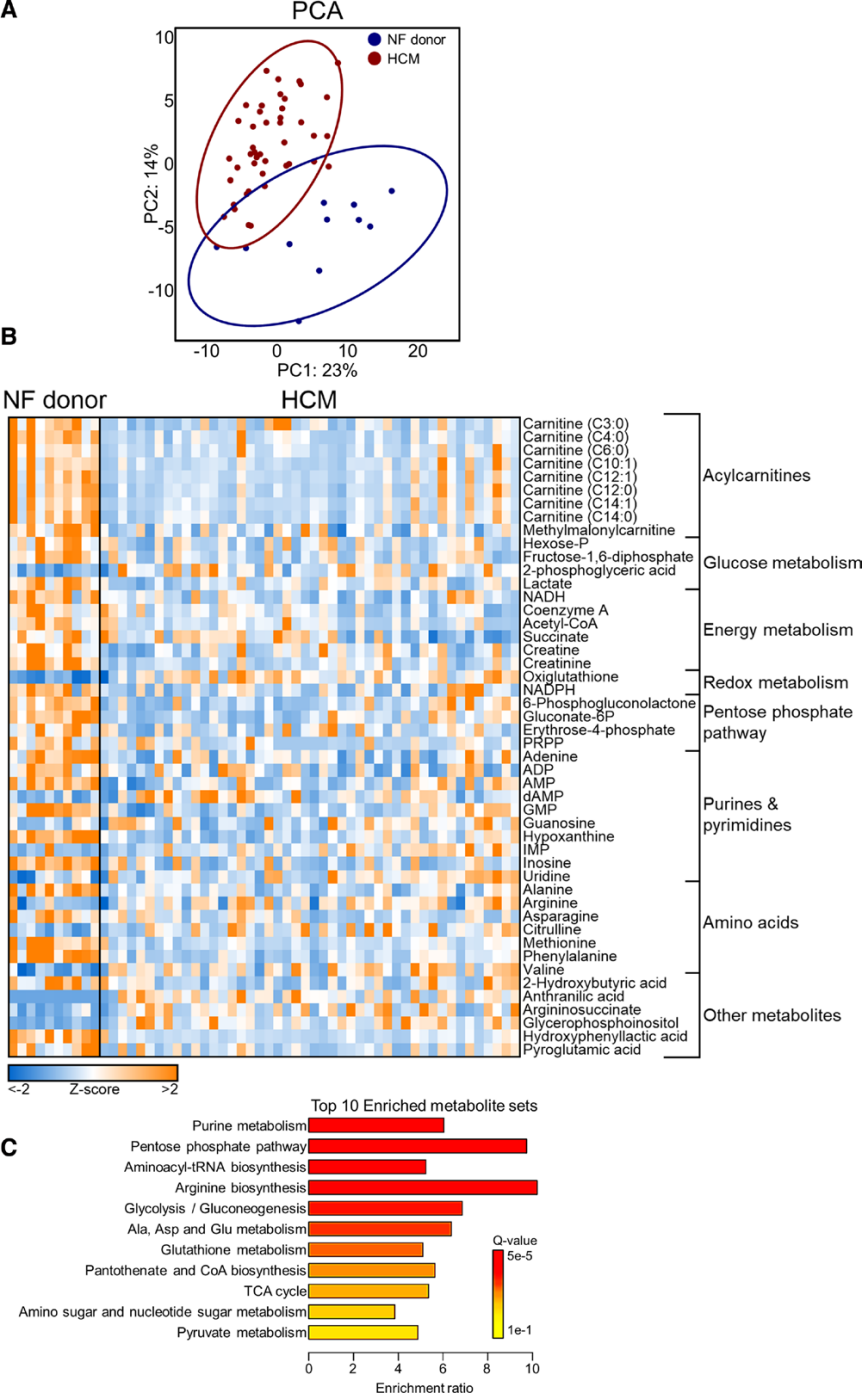
**Overview of metabolomic differences between hypertrophic cardiomyopathy (HCM) myectomy tissue and cardiac tissue from nonfailing (NF) donors. A**, Principal component analysis (PCA) showing distinct clustering of patients with HCM and NF donors. **B**, Heatmap showing *z* scores of all significantly different metabolites in HCM vs NF donor hearts (Q<0.05). **C**, Top 10 enriched metabolite sets in HCM vs NF donors analyzed via MetaboAnalyst 5.0.^18^ dAMP indicates deoxyadenosine monophosphate; Gluconate-6P, gluconate-6-phosphate; GMP, guanosine monophosphate; Hexose-P, hexose-phosphate; IMP, inosine monophosphate; NADH, reduced nicotinamide adenine dinucleotide; NADPH, reduced nicotinamide adenine dinucleotide phosphate; PRPP, phosphoribosyl pyrophosphate; and TCA, tricarboxylic acid.

We performed metabolomics on myectomy samples from patients with HCM (N=47) and nonfailing donor cardiac samples (N=12) to characterize the metabolite profile in HCM. Metabolomic differences between nonfailing donors and patients with HCM are shown in Figure 1. In total, 141 metabolites were detected, 51 of which were differently abundant in HCM versus nonfailing donors (Q<0.05). HCM and nonfailing donor samples displayed separate clustering on principal component analysis (Figure 1A), indicating clearly different metabolite profiles. G_positive_ and G_negative_ HCM samples did not cluster separately (Figure S2A), and no metabolites were significantly different between these groups. A heatmap of all statistically significant different metabolites in HCM versus nonfailing donors is shown in Figure 1B. Multiple acylcarnitines were less abundant in HCM versus nonfailing donors. We additionally observed altered levels of several glycolytic intermediates and a reduced abundance of metabolites involved in energy metabolism. Oxidized glutathione was elevated, while nicotinamide adenine dinucleotide phosphate was lowered in HCM versus nonfailing donors, indicative of oxidative stress. Major changes were also seen in metabolites linked to biosynthetic pathways such as the pentose phosphate pathway and purine/pyrimidine metabolism. Numerous amino acids were furthermore altered in HCM versus nonfailing donors. Accordingly, the most significantly enriched metabolic pathways were purine metabolism, the pentose phosphate pathway, and biosynthesis of tRNAs and arginine (Figure 1C). Altogether, metabolomic profiling in HCM yields a marked downregulation of fatty acid oxidation, while changes in glycolytic and biosynthetic pathways were rather bidirectional.

### Decreased Fatty Acid Storage and No Ceramide-Induced Lipotoxicity in HCM Despite Downregulation of Fatty Acid Oxidation

**Figure 2.**
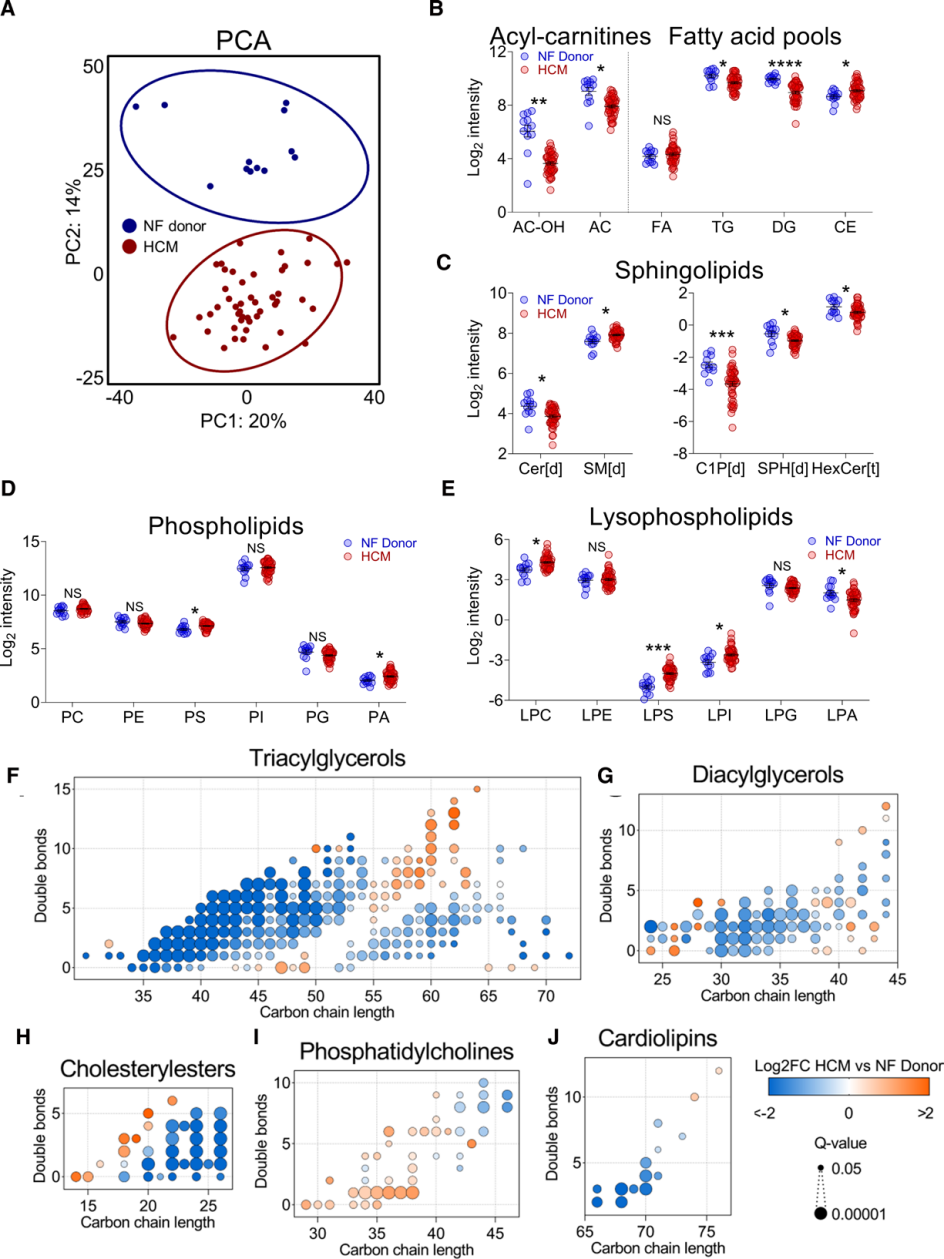
**Overview of lipidomic differences between hypertrophic cardiomyopathy (HCM) myectomy tissue and cardiac tissue from nonfailing (NF) donors. A**, Principal component analysis (PCA) showing distinct clustering of patients with HCM and NF donors. **B** through **E**, log2-transformed data for total amounts of lipids within a class, grouped per theme. Abbreviations of lipid classes are detailed in Table S3. **F** through **J**, Major alterations in individual lipid species abundance within a lipid class as dot plots, in which each dot represents a significantly altered lipid species. Vertical axes indicate double bond number, and horizontal axes indicate the carbon chain length of lipid species. The color and size of dots indicate fold change (FC) between groups and the significance of these changes, respectively.

To identify changes in lipid biology in HCM, we analyzed lipid profiles in HCM myectomy samples (N=47) and nonfailing donor cardiac samples (N=12) via lipidomics. Lipidomic observations are displayed in Figure 2. A total of 2139 lipids were detected, divided over 55 lipid classes (see Table S3 for an overview of abbreviations). Principal component analysis revealed markedly distinct clustering of HCM and nonfailing donor samples (Figure 2A). No differences were observed between G_positive_ and G_negative_ patients (Figure S2B). Consistent with metabolomic data, we observed markedly lowered levels of acylcarnitines in HCM versus nonfailing donors, which is in good agreement with previous work reporting downregulation of fatty acid oxidation in HCM^10–13,15^ (Figure 2B). Interestingly, despite defective fatty acid oxidation capacity, we observed unchanged levels of free fatty acids and less storage of fatty acids in major storage depots (ie, diacylglycerol and triacylglycerol). Levels of lipotoxic ceramides and associated sphingolipids ceramide-1-phosphate, sphingosine, and hexosyl-ceramide were furthermore reduced in HCM versus nonfailing donors, suggesting that fatty acids in HCM hearts were not directed more toward ceramide formation despite diminished oxidation and storage. Sphingomyelins were however more abundant in HCM versus nonfailing donors, indicating that excess fatty acids may be preferably routed toward this class of sphingolipids in HCM hearts (Figure 2C). Changes in terms of phospholipids included elevation of both phosphatidylserine and lysophosphatidylserine, higher levels of phosphatidic acid but lower levels of lysophosphatidic acid, and increased abundance of lysophosphatidylcholine and lysophosphatidylinositol in HCM versus nonfailing donors (Figure 2D and 2E). Whereas total triacylglycerol levels were reduced in HCM versus nonfailing donors, we observed an elevation of triacylglycerol species with relatively long and less saturated acyl chains (Figure 2F). Regarding the diacylglycerol pool, particularly medium-length species were depleted (Figure 2G). While the total pool of cholesteryl esters was increased, the majority of these species were actually less abundant, and a shift toward shorter chain cholesteryl esters was observed in HCM versus nonfailing donors (Figure 2H). Phosphatidylcholines were in addition relatively shorter and more saturated in HCM versus nonfailing donor hearts (Figure 2I). Total levels of the mitochondrial membrane–specific phospholipid cardiolipin were unchanged; however, we did observe an apparent shift from short, saturated cardiolipin toward longer, more unsaturated cardiolipin species in HCM (Figure 2J).

### In-Sequence Proteomics Yields Downregulation of Mitochondrial Translation and Increased Expression of Proteasome Proteins as Novel HCM Disease Features

**Figure 3.**
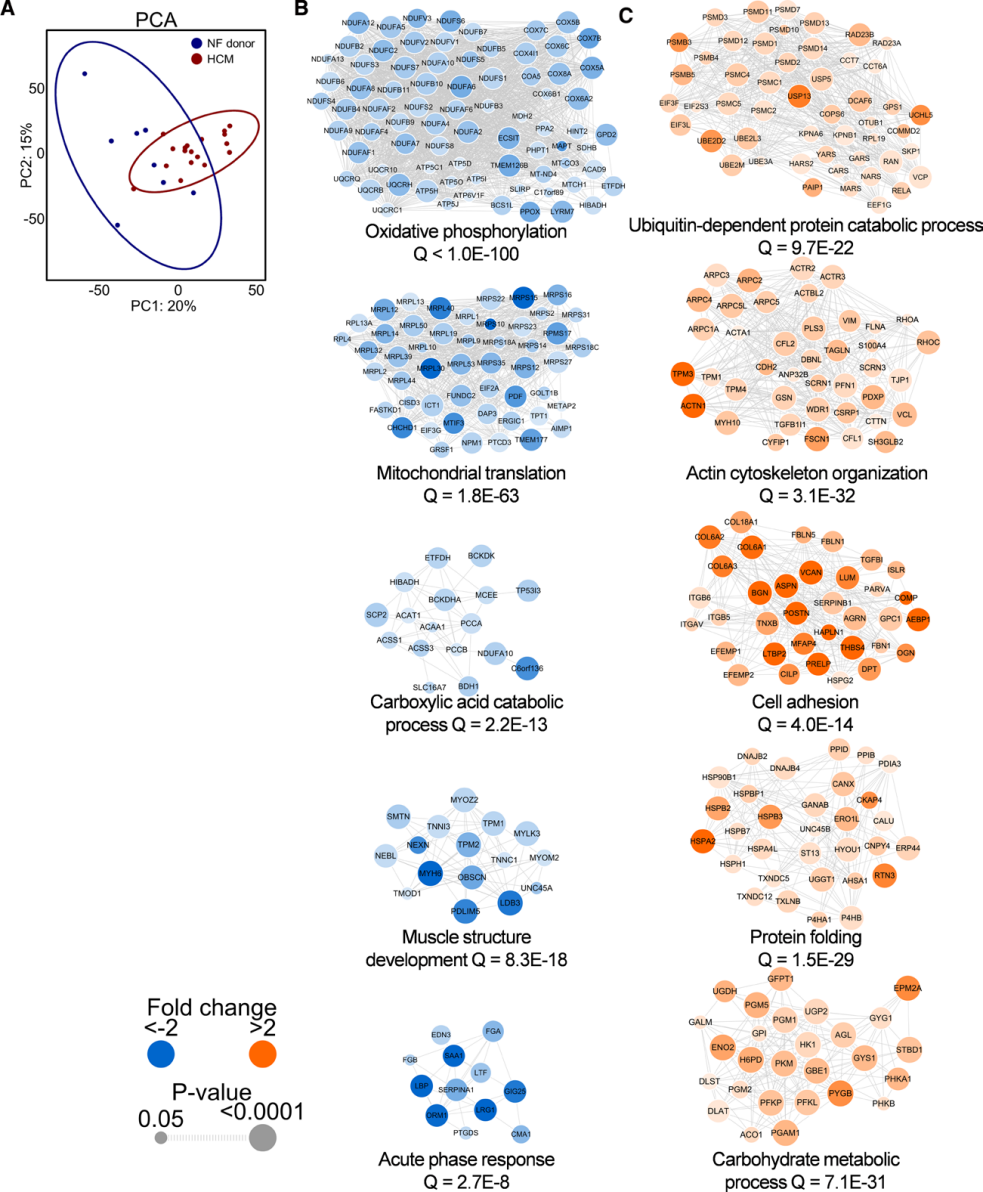
**Summary of proteomic differences between hypertrophic cardiomyopathy (HCM) and nonfailing (NF) donors. A**, Principal component analysis (PCA) showing distinct clustering of patients with HCM and NF donors. Top 5 downregulated clusters of interacting proteins (**B**) and the top 5 upregulated clusters of interacting proteins (**C**) according to cluster size. The color and size of protein nodes indicate fold change between groups and the significance of these changes, respectively.

Proteomics was performed on HCM myectomy samples (N=18) and nonfailing donor cardiac samples (N=8). An overview of proteomic findings is shown in Figure 3. To achieve maximum coverage of myocardial proteins, we performed in-sequence mass spectrometry on cytosolic-, myofilament-, and membrane-enriched fractions. In total, 3258 proteins were detected. HCM samples clustered separately from nonfailing donor samples (Figure 3A); G_positive_ and G_negative_ HCM samples did not differ from one another (Figure S2C). To identify downregulated and upregulated cellular processes in HCM versus nonfailing donor hearts, we performed gene ontology analysis on clusters of interacting proteins, as per the STRING database.^25^ Observations consistent with previous reports included lower levels of oxidative phosphorylation proteins, upregulation of proteins associated with the actin- and tubulin cytoskeleton (Figure S3), elevated deposition of extracellular matrix (ie, cell adhesion) proteins, and activation of protein quality control (ie, protein folding) in HCM versus nonfailing donor hearts (Figure 3B and 3C).^9,10,12,13,26^ We also report a marked reduction of proteins involved in mitochondrial translation in HCM versus nonfailing donors, many of which are components of the mitochondrial ribosomal machinery (Figure 3B), suggesting an overall impairment of mitochondrial protein synthesis in HCM. Additionally, we observed a higher abundance of proteins involved in ubiquitin-dependent proteasomal protein degradation (Figure 3C) in HCM versus nonfailing donors. Taken together, using high-coverage proteomics, we confirm key cellular hallmarks of HCM and identify downregulation of mitochondrial translation and upregulation of proteasome proteins as novel factors implicated in HCM pathophysiology.

### Integration of Multiomics Data Unveils Complex Alterations in Energy Metabolism Pathways

**Figure 4.**
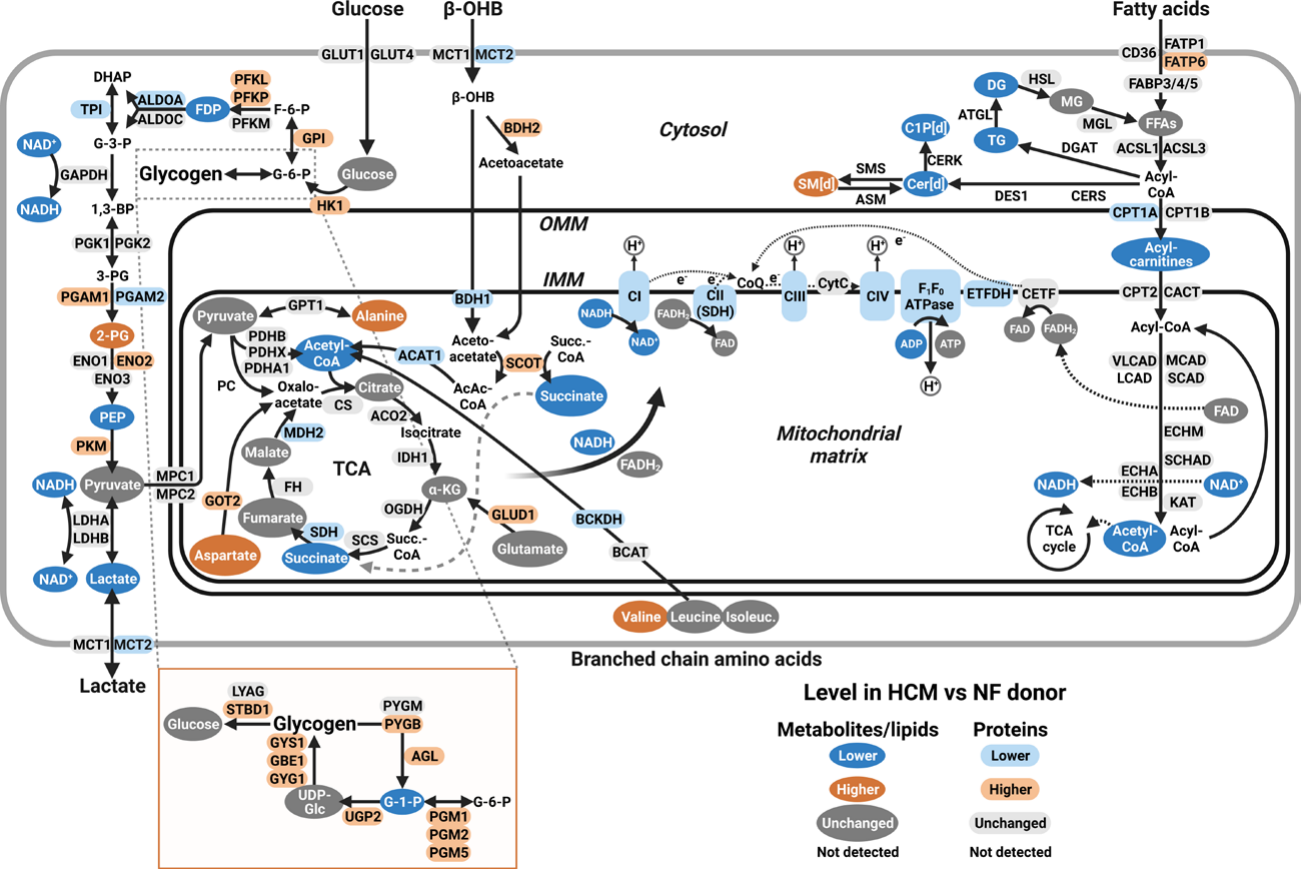
**Comprehensive overview of alterations in energy metabolism and lipid-handling pathways in patients with hypertrophic cardiomyopathy (HCM) vs nonfailing (NF) donors integrating metabolomics, lipidomics, and proteomics data.** Metabolomic, lipidomic, and proteomic data were integrated to provide an overview of changes in major energy metabolism and lipid-handling pathways. Metabolites and lipids are indicated by objects with a dark fill and white text; proteins are indicated by objects with a light fill and black text. Blue, orange, and gray indicate lower, higher, and unchanged abundances in HCM vs NF donors, respectively. Created with BioRender.com.

To comprehensively map alterations in lipid-handling and energy metabolism pathways, we integrated proteomic, metabolomic, and lipidomic data (Figure 4). Surprisingly, despite the downregulation of fatty acid oxidation in HCM, the vast majority of proteins involved in fatty acid uptake and subsequent shuttling to the mitochondria and β-oxidation were unchanged in HCM versus nonfailing donors. Only 1 protein of the carnitine shuttle (CPT1A [carnitine palmitoyltransferase I]) and ETFDH (electron transferring flavoprotein dehydrogenase) that feeds electrons from β-oxidation-derived FADH_2_ into the electron transfer system were less abundant in HCM versus nonfailing donors. This suggests that impaired fatty acid oxidation capacity in HCM is not primarily regulated by protein abundance. In terms of glucose metabolism, we found that the majority of proteins involved in glycolysis were more abundant in HCM versus nonfailing donors. This was accompanied by decreased levels of fructose-1,6-diphosphate and phosphoenolpyruvate and higher levels of 2-phosphoglycerate. Interestingly, we also found a global upregulation of proteins involved in glycogen metabolism (eg, glycogen synthetase, glycogen phosphorylase), but it was unclear whether these data would predict accumulation or depletion of myocardial glycogen levels. We measured glycogen amounts in cardiac tissue from patients with HCM collected at the time of myectomy and explanted tissue from patients with HCM with end-stage heart failure to assess disease stage–specific levels of glycogen. Compared with nonfailing donors, we observed no myocardial accumulation of glycogen in patients with HCM at any disease stage (Figure S4A). There were also no differences in myocardial glycogen levels between G_negative_ and G_positive_ patients at the time of myectomy (Figure S4B). Thus, higher levels of proteins involved in glycogen metabolism without altered glycogen levels may suggest increased glycogen synthesis and breakdown in HCM. Lower lactate abundance suggests that anaerobic glycolysis was not increased in HCM versus nonfailing donors and may possibly indicate increased usage of lactate to fuel pyruvate production, which has been observed in failing hearts.^27^ Acetyl-CoA levels were lower in HCM versus nonfailing donors; however, we found no evidence of downregulation of pyruvate to acetyl-CoA conversion. Consequently, lower acetyl-CoA levels may particularly result from impaired acetyl-CoA input from mitochondrial fatty acid oxidation. While most tricarboxylic acid (TCA) cycle enzymes and intermediates were unchanged, it appears that acetyl-CoA conversion to citrate is maintained to prevent TCA intermediate depletion. Additionally, we observed an increased abundance of several proteins that participate in anaplerosis, replenishing TCA intermediates via transamination of amino acids (ie, GOT2 [aspartate aminotransferase], GLUD1 [glutamate dehydrogenase 1]), raising the possibility that amino acids may be alternative substrates to fuel the TCA cycle. Catabolism of branched-chain amino acids, however, appeared to be blunted, which has also been reported in other settings of heart failure.^28^ We found conflicting observations with respect to ketone oxidation. Cytosolic BDH (3-hydroxybutyrate dehydrogenase) 2 and SCOT (succinyl-CoA-3-oxaloacid CoA transferase) were elevated, while mitochondrial BDH1 and ACAT1 (acetyl-CoA acetyltransferase 1) were less abundant in HCM. Thus, it remains unclear whether ketones are differentially used in HCM hearts. In summary, altered energy metabolism in HCM is typified by (1) downregulation of fatty acid oxidation without overtly lowered levels of proteins involved in this pathway; (2) generally higher abundance of glycolysis and glycogen turnover–associated proteins, which may suggest increased usage of glucose as energy fuel; and (3) no indications of impaired pyruvate entry into the TCA cycle and no hints toward increased anaerobic conversion of pyruvate to lactate.

### Cardiac Remodeling Is Differentially Associated With Metabolite Levels in G_negative_ and G_positive_ Patients

**Figure 5.**
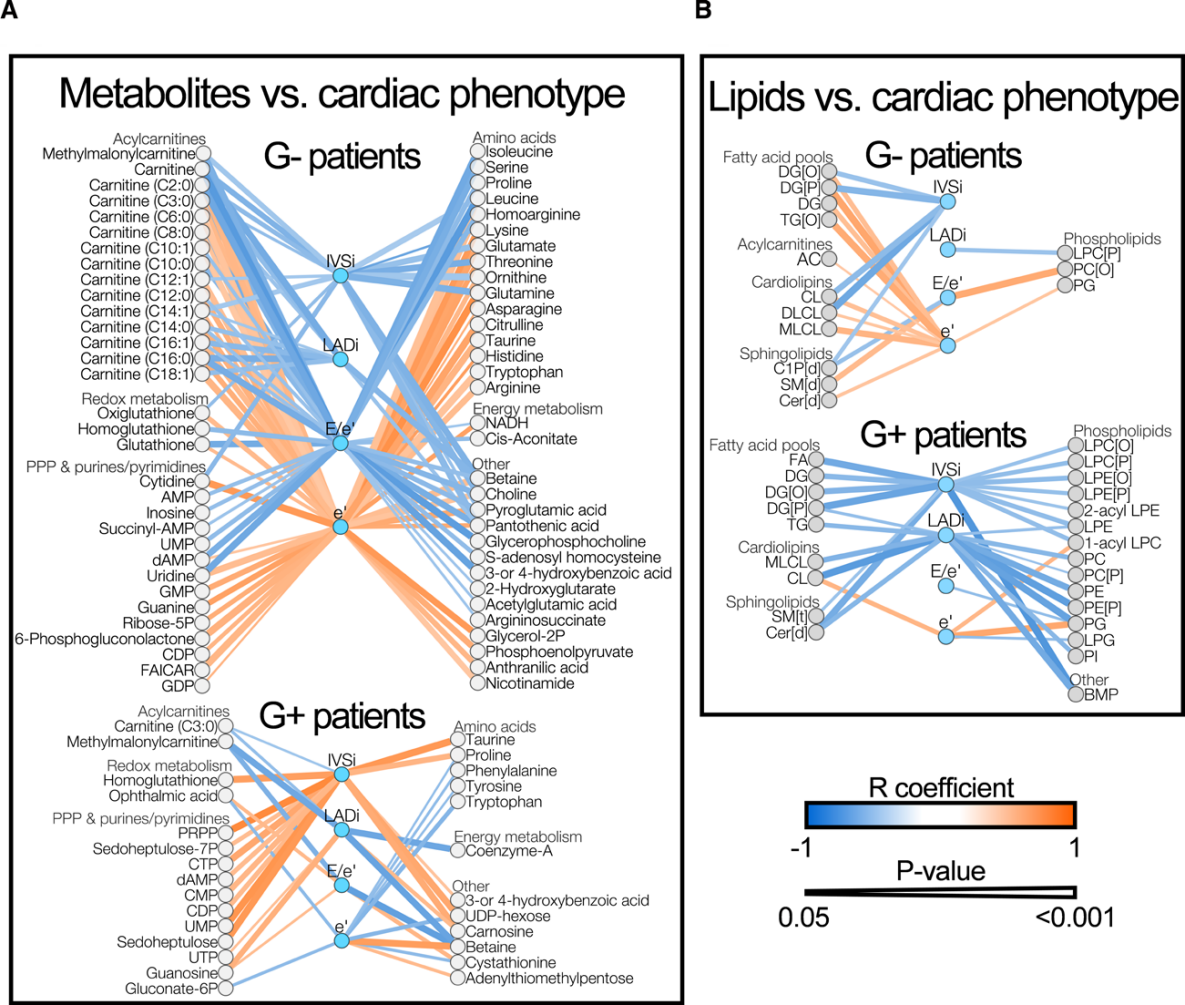
**Correlations between cardiac phenotype and metabolites and lipids in patients with hypertrophic cardiomyopathy (HCM).** Correlations were tested via linear regression and are described by the Pearson R coefficient. **A**, All significant correlations between cardiac parameters and metabolites in genotype-negative (G_negative_; G−) and genotype-positive (G_positive_; G+) patients. **B**, All significant correlations between cardiac parameters and lipids classes in G_negative_ and G_positive_ patients. Blue and orange lines indicate negative and positive correlations, respectively. Line thickness corresponds with the significance level of the correlation. Exact R coefficients and *P* values, as well as group comparison statistics between nonfailing donors and G− or G+ patients of metabolites and lipids in these correlations, are available as Supplemental Material (Correlation statistics). IVSi indicates septal thickness indexed to body surface area; and LADi, left atrial diameter indexed to body surface area.

We previously observed that mitochondrial dysfunction was tightly linked to septal hypertrophy in G_negative_ HCM but not in G_positive_ HCM,^15^ which suggests that also metabolic alterations may correlate to cardiac remodeling differentially in these patient groups. Therefore, we explored how metabolites and lipids were associated with parameters of cardiac function in G_negative_ and G_positive_ patients with HCM (Figure 5). Strikingly, in G_negative_ patients, we observed that numerous acylcarnitines were negatively associated with septal hypertrophy, atrial dilatation, and diastolic dysfunction. In contrast, we did not observe such a clear pattern in G_positive_ patients, in whom only 2 acylcarnitines negatively correlated with pathological cardiac remodeling. These findings are in line with the notion that particularly in G_negative_ HCM, cardiac remodeling is intertwined with impaired bioenergetic homeostasis.^15^ Another remarkable discrepancy was seen with respect to metabolites involved in the pentose phosphate pathway and downstream synthesis of purines and pyrimidines, which constitute the building blocks of RNA and DNA. In G_negative_ patients, these metabolites were negatively correlated to diastolic dysfunction, whereas in G_positive_ patients, these metabolites displayed strong positive associations with septal hypertrophy and modest positive correlations with diastolic impairment. Similarly, in G_negative_ samples, a vast array of amino acids showed negative associations with septal hypertrophy and diastolic dysfunction, whereas in G_positive_ patients, several amino acids were positively associated with septal hypertrophy and negatively with diastolic function. These findings suggest that in G_negative_ HCM, the progression of cardiac disease is associated with the depletion of metabolites that are necessary for the synthesis of proteins and nucleosides, while in G_positive_ HCM, septal hypertrophy is accompanied by elevated levels of such metabolites. Finally, several metabolites involved in reactive oxygen species defense (ie, redox metabolites) were negatively correlated with septal hypertrophy and diastolic dysfunction in G_negative_ patients, while in G_positive_ patients, such metabolites were positively associated with adverse cardiac changes. This may suggest that oxidative stress is differently involved in disease progression in G_negative_ and G_positive_ HCMs.

Regarding associations between lipids and cardiac phenotype, we did not observe drastic differences between G_negative_ and G_positive_ patients with HCM (Figure 5B). Remarkably, in both patient groups, we nearly exclusively observed negative associations between lipid amounts and pathological cardiac alterations. In G_positive_ patients, many lipid levels were inversely linked to septal hypertrophy and atrial dilatation. This included free fatty acids and fatty acid storage depots (ie, diacylglycerol, triacylglycerol) and lipotoxic ceramides. G_negative_ patients displayed similar albeit less pronounced patterns, characterized by diastolic compliance being positively linked to diacylglycerol and ceramide levels. Thus, in both patient groups, cardiac hypertrophy and functional impairment were accompanied by reduced fatty acid storage and ceramide levels.

In previous work, we measured mitochondrial respiratory parameters in fresh myectomy samples from the same patients whose myectomy samples were analyzed in the present study.^15^ We, therefore, also explored correlations between mitochondrial respiratory function and metabolites and lipids (Figure S5). In G_negative_ patients, we observed numerous positive associations between mitochondrial respiratory parameters on the one hand and acylcarnitines, redox metabolites, PPP and purine/pyrimidine metabolites, amino acids, and metabolites involved in energy metabolism on the other hand (Figure S5A). These metabolites were negatively associated with septal hypertrophy and diastolic impairment in G_negative_ patients (Figure 5), thus suggesting that in G_negative_ HCM, pathological cardiac remodeling and functional impairment are interwoven with fatty acid oxidation impairment, mitochondrial respiratory dysfunction, impaired antioxidant defense, and reduced levels of biosynthetic metabolites. In contrast, G_positive_ samples displayed only few associations between metabolites and mitochondrial function (Figure S5B), which is consistent with the observation that mitochondrial impairment in G_positive_ HCM is not linked to cardiac remodeling.^15^ By extension, in samples from G_negative_ patients, but not G_positive_ patients, mitochondrial respiration was overall positively associated with lipids that displayed negative associations with cardiac hypertrophy and diastolic dysfunction.

**Figure 6.**
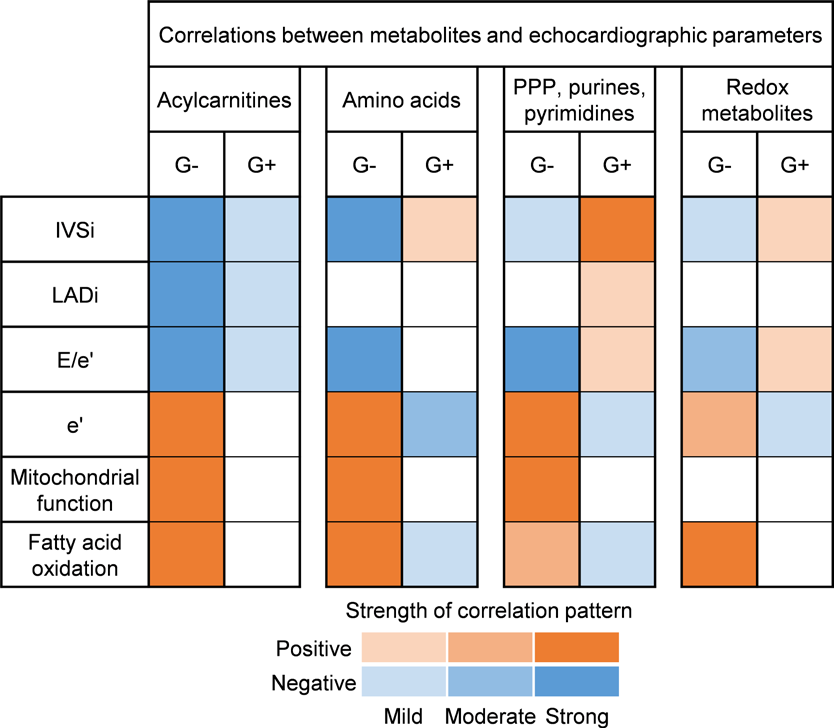
**Contrasting metabolic alterations underlie cardiac remodeling in genotype-negative (G−) and genotype-positive (G+) patients with hypertrophic cardiomyopathy (HCM).** Matrix summarizing correlation patterns between metabolite classes and (functional) cardiac parameters in G− and G+ patients with HCM. Negative and positive correlation patterns are colored blue and orange, respectively. The color intensity reflects the strength of correlation patterns. IVSi indicates interventricular septum thickness indexed to body surface area; LADi, left atrial diameter indexed to body surface area; and PPP, pentose phosphate pathway;

Combined, by linking echocardiographic and mitochondrial respiration data to metabolomic and lipidomic data, we observed markedly different correlation patterns in G_negative_ and G_positive_ patients with HCM, as summarized in Figure 6. G_negative_ patients displayed an overall connectedness between cardiac remodeling, mitochondrial dysfunction, fatty acid oxidation impairment, and lowering of acylcarnitines, biosynthetic metabolites, and redox metabolites. Conversely, in G_positive_ patients, both biosynthetic metabolites and redox metabolites were positively associated with cardiac remodeling. Collectively, these observations imply major pathophysiological differences between G_negative_ and G_positive_ patients with HCM.

## DISCUSSION

**Figure 7.**
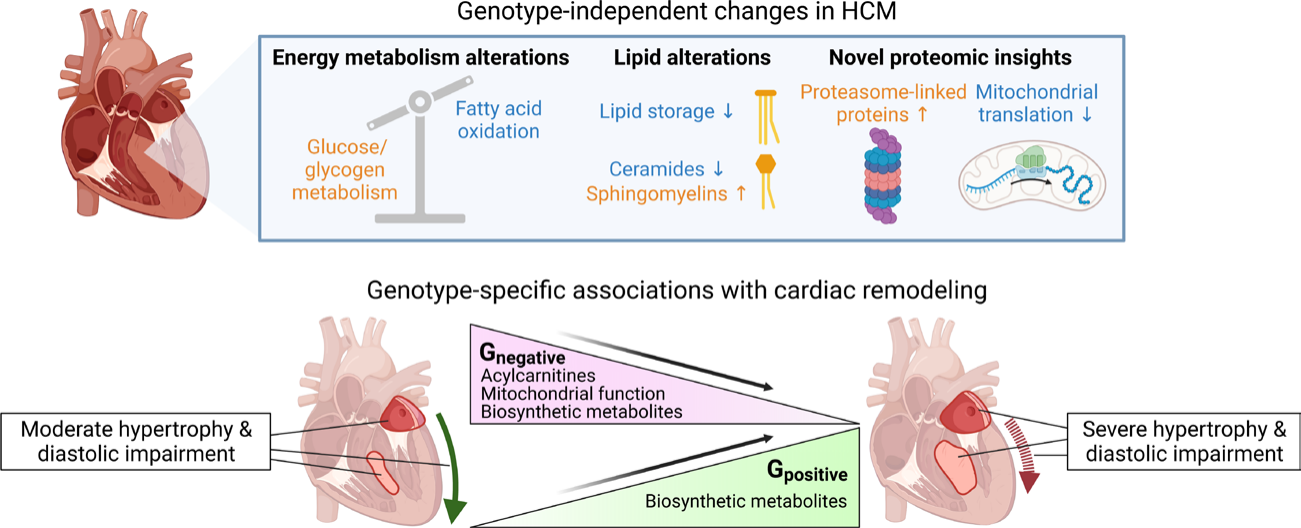
**Summary of the main findings of this study.** Genotype-independent changes in hypertrophic cardiomyopathy (HCM) included downregulation of fatty acid oxidation and increased glucose metabolism and glycogen turnover; decreased lipid storage and ceramide formation and increased sphingomyelin abundance; and proteasome upregulation and downregulation of mitochondrial translation proteins. G_negative_ patients with HCM displayed negative associations between cardiac remodeling and fatty acid oxidation, mitochondrial function, and levels of biosynthetic metabolites (ie, pentose phosphate pathway [PPP] intermediates, purines, pyrimidines, and amino acids). Conversely, G_positive_ patients with HCM displayed positive associations between biosynthetic metabolites and cardiac remodeling. Thus, opposite metabolic changes are linked to cardiac disease in G_positive_ and G_negative_ patients. Created with BioRender.com.

To identify general and genotype-specific metabolic alterations in HCM, we conducted multiomics analyses on myectomy samples from a well-phenotyped cohort of patients with HCM and cardiac tissue from age- and sex-matched and body mass index–matched nonfailing donors. The main findings of our study are summarized in Figure 7. Our data suggest that common features of HCM are (1) a shift away from fatty acid oxidation toward glucose metabolism and glycogen turnover; (2) reduced lipid storage and ceramide formation, but elevated sphingomyelin levels; and (3) upregulation of proteins involved in proteasomal protein degradation and downregulation of mitochondrial translation machinery. In G_negative_ patients with HCM, cardiac remodeling was associated with impaired fatty acid oxidation, mitochondrial dysfunction, and lower abundance of biosynthetic metabolites (ie, PPP intermediates, purines, pyrimidines, and amino acids) and redox metabolites. In G_positive_ patients with HCM, biosynthetic metabolites and redox metabolites were positively associated with cardiac remodeling. These findings indicate contrasting metabolic alterations underlie cardiac disease in G_positive_ and G_negative_ patients.

### Proteasome Upregulation and Reduced Mitochondrial Translation as Novel Features of HCM

Using high-coverage in-sequence proteomics, we here identified upregulation of proteasome-linked proteins and downregulation of mitochondrial translation proteins as novel hallmarks in HCM, irrespective of genotype. Impairment of the ubiquitin-dependent proteasome system has been observed in HCM mouse models and human HCM myocardium,^29,30^ which has been linked to oxidative damage of proteasomal subunits.^30,31^ Our analyses indicate that proteasome malfunctioning is unlikely to stem from the depletion of proteasomal machinery and raise the question of whether the observed upregulation of proteasomal subunits might reflect a compensatory response to functional impairment. We observed a marked reduction of proteins involved in mitochondrial translation, particularly mitochondrial ribosomal subunits, suggesting reduced mitochondrial protein synthesis in HCM. The involvement of altered mitochondrial translation in heart disease has not been extensively studied. Several studies report a link between mitochondrial ribosome protein gene mutations and cardiomyopathy.^32,33^ Gao et al^34^ recently demonstrated that knockout of MRPS5 (mitochondrial ribosomal protein S5) resulted in cardiac hypertrophy and heart failure, which was accompanied by mitochondrial dysfunction and metabolic remodeling. In contrast, *mrps-5* suppression extended lifespan in *Caenorhabditis* *elegans*,^35,36^ and reduction of mitochondrial protein synthesis in heterozygous *Mrpl54* mutant mice did not affect cardiac health.^37^ These studies show that manipulating mitochondrial ribosome function can incite both beneficial and detrimental effects, most likely related to dosage effects, and further study is warranted to elucidate the functional consequences of reduced mitochondrial ribosomal protein levels in HCM.

### Energy Metabolism and Lipid Alterations in HCM

By integrating multiomics data, we comprehensively mapped alterations in energy metabolism pathways. Consistent with elevated myocardial glucose uptake in patients with HCM,^38^ we found that the majority of proteins involved in glycolysis were increased in HCM versus nonfailing donor samples. This did not seem to be accompanied by increased anaerobic glycolysis, as lactate levels were lower in HCM samples. Glucose did also not seem to be stored excessively as glycogen, which even appeared to be reduced at end-stage HCM. Thus, although glycogen storage diseases may manifest as HCM phenocopy,^39^ glycogen storage is not a general feature of HCM. These observations are discordant with previous work reporting elevated glycogen deposition in myocardium of patients with HCM displaying impaired contractile reserve.^40^ Importantly, that study did not differentiate patients with HCM based on genotype; thus, it is unknown whether that study cohort included patients with glycogen storage diseases.

Pyruvate levels and pyruvate-derived TCA input did not seem abnormal in HCM versus nonfailing donors, suggesting that oxidative glucose metabolism was sustained in HCM. Surprisingly, despite the reduced use of fatty acids as energy substrate in HCM, the only proteins involved in fatty acid oxidation that were less abundant were CPT1A and ETFDH. While downregulation of these proteins may impair acylcarnitine shuttling and electron input into the electron transfer system, respectively, it is likely that other factors than enzyme abundance (eg, posttranslational modifications of fatty acid oxidation enzymes^41^) contribute substantially to the downregulation of mitochondrial fatty acid oxidation in HCM. In other heart failure etiologies, impaired fatty acid oxidation capacity is typically met with a mismatch in lipid uptake, causing accumulation of free fatty acids and lipotoxic ceramides and increased lipid storage.^42,43^ We did not observe this and even found that fatty acids, diacylglycerols, triacylglycerols, and ceramides were negatively associated with cardiac remodeling, which is consistent with previous work showing a global reduction of lipid levels in advanced heart failure tissue.^44^ Notably, in G_negative_ patients, ceramide and ceramide-1-phosphate levels were positively correlated with fatty acid oxidation capacity. Altogether, these observations suggest that during cardiac remodeling in HCM, no overt mismatch between fatty acid uptake and oxidation occurs. Instead, we put forward that reduced use of fatty acids as fuel is orchestrated in concert with reduced lipid uptake and storage. This would be in line with our observation of numerous phospholipids being negatively associated with cardiac remodeling, as phospholipid synthesis requires fatty acids. Previous work provided indications of reduced fatty acid uptake and decreased CD36 expression in nongenotyped HCM.^45^ However, in our hands, the levels of fatty acid transport proteins were unchanged; thus, it would be pertinent to study whether the expression and activity of CD36 in HCM with well-characterized genotype are altered due to posttranslational modifications and changes in localization.^46^

### Divergent Associations Between Cardiac and Metabolic Remodelings in G_positive_ and G_negative_ HCMs

We observed contrasting patterns of correlations between cardiac remodeling and metabolite levels in G_negative_ and G_positive_ patients. In G_negative_ patients, cardiac remodeling was associated with mitochondrial dysfunction and lowering of acylcarnitines and biosynthetic metabolites (ie, PPP intermediates, purines, pyrimidines, and amino acids). The energetic cost of production of purines and pyrimidines via the PPP and protein synthesis from amino acids is substantial^47,48^; thus, reductions in the levels of metabolites related to these processes are to be expected when bioenergetic homeostasis is compromised as a consequence of mitochondrial dysfunction. The added hemodynamic burden from left ventricle outflow tract obstruction further deteriorates cardiac function in obstructive HCM^49^ and is likely to exacerbate the already high myocardial energy consumption.^7,8^ Consistent with previous studies, the obstruction of the left ventricle outflow tract is more pronounced in G_negative_ patients (Table S1).^5,24^ Our observations reinforce the notion that G_negative_ HCM is a condition driven by energetic and metabolic impairment^15^ although the primary cause of disease remains unknown. Further study is warranted to assess how the downregulation of biosynthetic processes may contribute to disease progression in G_negative_ HCM. In contrast to G_negative_ patients, G_positive_ patients displayed positive associations between cardiac remodeling and biosynthetic metabolites. These observations may suggest that in G_positive_ HCM, biosynthesis of nucleotides and proteins is upregulated to facilitate cardiac hypertrophy.^50^ Wang et al^13^ proposed that interfering with the PPP might represent a therapeutic strategy in HCM. Based on our observations, such a strategy may indeed theoretically inhibit hypertrophy in G_positive_ HCM by reducing the availability of nucleotides needed to promote cardiac growth but would be inadvisable in G_negative_ HCM, as, in these patients, loss of PPP, purine, and pyrimidine intermediates was intertwined with cardiac remodeling. Taken together, our findings clearly suggest that different patterns of metabolic alterations underpin cardiac remodeling in G_negative_ and G_positive_ HCM, and future research is needed to gain mechanistic insight.

### Lack of Consensus Among Studies Into Metabolomic and Lipidomic Landscapes in HCM

**Figure 8.**
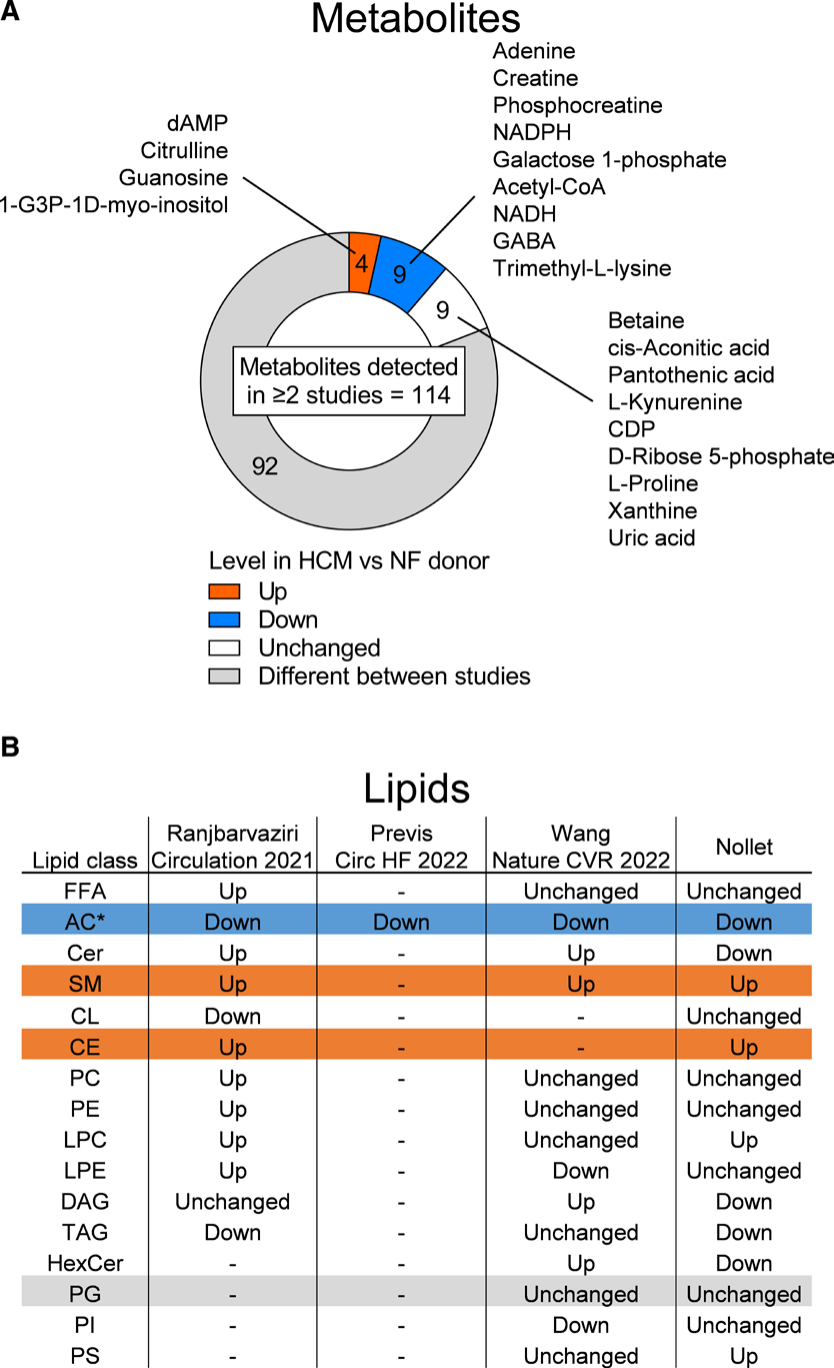
**Limited agreement among studies into metabolomic and lipidomic changes in tissue of patients with hypertrophic cardiomyopathy (HCM) compared with nonfailing (NF) donor tissue. A**, Pie chart visualizing agreement in metabolite findings in HCM vs NF donor cardiac tissue samples; 114 metabolites were detected in at least 2 studies. Of these metabolites, 92 displayed inconsistent outcomes in HCM vs NF donor tissue. **B**, Table displaying consistency in lipidomic findings in HCM vs NF donor tissue. Blue and orange indicate consistently lowered and elevated levels, respectively, in HCM vs NF donors. *Derived from both metabolomics and lipidomics.

Three other recent studies have aimed to define the lipidome and metabolome in cardiac tissue of patients with obstructive HCM relative to nonfailing donor tissue.^11–13^ An overview of patient and nonfailing donor demographics in these studies and the present study is shown in Table S4. To map the degree of agreement between studies, we visualized the extent to which metabolomic and lipidomic data were consistent (Figure 8). In total, 114 metabolites were detected in at least 2 studies (Figure 8A; Table S5). Twenty-two of these metabolites displayed similar behavior in HCM versus nonfailing donor tissue. Depletion of acetyl-CoA, phosphocreatine, nicotinamide adenine dinucleotide, and nicotinamide adenine dinucleotide phosphate was consistently observed, suggesting that energetic impairment is a robust feature of HCM. However, the vast majority of metabolomic findings differed between studies. Similarly, lipidomic findings were mostly different between studies, only showing consistent reductions in acylcarnitines and elevations in sphingomyelins and cholesteryl esters in HCM versus nonfailing donors (Figure 8B). These discrepancies suggest that the external validity of group differences observed between nonfailing donors and patients with HCM is relatively limited. Possible explanations underlying this poor generalizability may include cohort differences, such as the degree of cardiac remodeling at the time of myectomy, the demographic and genetic makeup of the HCM patient study population, and the presence of comorbidities and medication use. Regarding comorbidities, average body mass index differed substantially between patient cohorts (Table S4), and it is unclear to what extent this might have influenced results in previous studies.^11–13^ With respect to medication usage, in our study, a substantial number of patients received antihypertensive drugs, suggesting the presence of hypertension in these patients, potentially modifying our findings. Usage of antihypertensive drugs was, however, not associated with overt hypertension (Figure S6). Moreover, omitting samples from patients on antihypertensive drugs from our correlation analyses (Figure S7) yielded correlation patterns consistent with Figure 5. Thus, the usage of antihypertensive drugs in our patient cohort did not drastically influence our observations. In conclusion, we argue that the involvement of metabolic and lipid alterations in HCM pathophysiology is more optimally identified by linking metabolomic and lipidomic data to variability in clinical patient data. This approach enabled us to identify G_negative_ and G_positive_ patient–specific associations between metabolic and cardiac remodeling and may serve as a valuable approach to unveil mutation type–specific associations as well when applied to larger cohorts.

### Study Limitations and Future Implications

We acknowledge several limitations of the study. First, the hearts of nonfailing donors were subjected to cold cardioplegia solution for a longer period of time than the hearts of patients, which may have masked or exacerbated some metabolic changes. Second, all patient samples were collected in the Netherlands, whereas nonfailing donor samples originated from Australia and the United States, which may potentially have introduced bias into our data. Third, given the observational nature of our study, we cannot infer causality of correlations. Nonetheless, our findings underscore that an integrated approach, where we combine clinical and experimental data, is a valuable approach to explore patient group–specific disease mechanisms. Establishing causality should be reserved for further research, utilizing mouse models and human stem cell–derived models of HCM. Our integrated multiomics data suggest an increase in glucose metabolism along with a downturn in fatty acid uptake and catabolism in HCM although our data do not allow for direct evaluation of substrate flux. Impaired fatty acid catabolism is in line with findings in a novel 2-hit mouse model of HCM, in which the onset of cardiomyopathy was associated with depressed fatty acid oxidation.^51^ A recent study found that failing human hearts with reduced ejection fraction surprisingly displayed retained capability of shifting energy metabolism toward fatty acid oxidation during intralipid infusion. This shift was accompanied by improved cardiac energetics and cardiac output,^52^ suggesting therapeutic benefit of improved myocardial delivery of fatty acids. Such a strategy could be equally effective as well in patients with HCM, given that cardiac remodeling appeared to be coupled to decreased fatty acid uptake.

### Conclusions

Metabolic remodeling in HCM was characterized by a shift away from fatty acid oxidation toward increased glucose and glycogen metabolism. Cardiac remodeling was associated with reduced ceramide formation and lipid storage. In G_negative_ patients, cardiac remodeling was linked to mitochondrial dysfunction and decreased levels of acylcarnitines and biosynthetic metabolites. Conversely, in G_positive_ patients, we observed positive associations between the abundance of biosynthetic metabolites and cardiac remodeling. Combined, our findings suggest that distinct metabolic alterations contribute to cardiac disease in G_positive_ and G_negative_ HCM.

## ARTICLE INFORMATION

### Acknowledgments

The authors are grateful to Georges Janssens (Amsterdam UMC, the Netherlands) for insightful discussions about the contents of this study.

### Sources of Funding

This work was supported by the Netherlands Organization for Scientific Research (NWO VICI; grant 91818602) and the Leducq Foundation (grant 20CVD01).

### Disclosures

Dr Margulies is a consultant for Bristol Myers Squibb and receives research support from Amgen.

### Supplemental Material

Supplemental Methods

Figures S1–S7

Tables S1–S5

Data Set Quality Data

Data Set Multi-Omics Data Sets

Data Set Correlation Statistics

References 53–63

## Supplementary Material


