# Supplementary material for: Integrating Clinical Phenotype With Multiomics Analyses of Human Cardiac Tissue Unveils Divergent Metabolic Remodeling in Genotype-Positive and Genotype-Negative Patients With Hypertrophic Cardiomyopathy
Source: Circ Genom Precis Med. 2024 Jun 10;17(3):e004369. doi: 10.1161/CIRCGEN.123.004369 (PMC11188634; doi:10.1161/CIRCGEN.123.004369)
Supplement: Supplementary file 1 [file hcg-17-e004369-s001.pdf]

SUPPLEMENTAL MATERIAL

Supplemental Methods

Human myocardial samples

Interventricular septal tissue from 47 patients with obstructive HCM (32 males, 15 females) was obtained during myectomy surgery and immediately snap-frozen and stored in liquid nitrogen. Of these patients, 41 underwent genetic testing as described previously [53]. Briefly, this entailed next generation sequencing using a panel of all known cardiomyopathy-related genes, including genes that are associated with HCM phenocopies. A sarcomere gene mutation was found in 19 patients (*i.e.* $G_{positive}$); no gene mutation was identified in 22 patients ($G_{negative}$), and 6 patients did not undergo genetic testing (Figure S1). Eleven LV cardiac tissue samples from end-stage failing HCM hearts were used for analysis of myocardial glycogen levels. These samples were obtained during heart transplantation and snap-frozen and stored in liquid nitrogen. LV tissue samples from 20 NF donors with no recorded history of cardiac disease were procured in accordance with protocols and ethical regulations approved by the Human Research Ethics Committee at the University of Sydney (Sydney, Australia; HREC Univ Sydney 2012/030) and Institutional Review Boards at the University of Pennsylvania and the Gift-of-Life Donor Program (Pennsylvania, USA). Hearts from NF donors were obtained during organ donation from brain-dead individuals. All hearts were arrested *in situ* using ice-cold cardioplegia solution and kept on ice until flash-freezing in liquid nitrogen within 4 hours of explantation as previously described [54,55]. Clinical and cardiac parameters of patients with obstructive HCM and NF donors are shown in Table S1. An overview of the study design and sample sizes per analysis are displayed in Figure S1. Table S2 offers detailed individual information of each patient and donor and indicates which samples were used for each method.

Proteomics

Sample preparation

Frozen myocardial tissue samples (20-50 mg) were fractionated into cytosolic protein-enriched fractions, myofilament protein-enriched fractions and membrane protein-enriched fractions according to the 'IN-Seq' method as described previously [19]. Protein concentrations were determined via the Bradford method. One hundred µg of each fraction was reduced using 1 mM tris(2-carboxyethyl)phosphine. Sample clean-up was performed using filter aided sample preparation (FASP 30 kDA, Promega, Madison, WI) as previously described [56]**.** Briefly, samples were digested for 15–18h at 37°C using ultra-grade Trypsin (Promega) at a 1:100 enzyme:protein ratio. Digestion was stopped by the addition of formic acid (0.1% vol/vol) final concentration. Samples were desalted using Oasis HLB plates (30 µm and 5 mg sorbent, Waters Milford, MA), vacuum dried and stored at -80°C until analysis.

Mass Spectrometry

Mass spectrometry (MS) data generation and MS raw spectra conversion to peptide and protein identification were performed blinded. Peptides from each patient sample were analyzed in three separate runs (one run each for cytosolic, myofilament and membrane protein-enriched fractions) by liquid chromatography-tandem mass spectrometry (LC-MS/MS) on a Dionex Ultimate 3000 NanoLC connected to an Orbitrap Elite (Thermo Fisher Scientific) equipped with an EasySpray ion source or on a Dionex Ultimate 3000 NanoLC connected to an Orbitrap Fusion™ Lumos™ Tribrid™ Mass Spectrometer (Thermo Fisher Scientific) equipped with an EasySpray ion source, following a previously published workflow [20]. Peptides were separated on a C18 column (15 cm with 300 µm ID, 3 µm Omega Polar C18 beads, and 100 Ǻ pore size, Phenomenex) over the course of 60 min at a flow rate of 7µL/min. Mobile phase A was composed of 0.1% vol/vol formic acid in water and mobile phase B of 90% vol/vol acetonitrile and 0.1% vol/vol formic acid in water. Initial loading condition was 1% B for 2 min and then 3% for another 3 min at a flow rate of 7 µL/min to allow for equilibration and for samples to reach the column, followed by a linear gradient of 3–28% B over 45 min, 28–45% B over 10 min. Each MS1 scan was followed by 40 data independent acquisition (DIA) MS2

scans (15 Da size precursor window). +1 and unassigned charge states were not selected for MS2 analysis. MS1 spectra were acquired at a resolution of full-width half-maximum 120.000 with a target setting of 400.000 ions and accumulation time of 50 ms. For MS2, 500.000 ions were accumulated in the Orbitrap over a maximum time of 30 ms and scanned at a resolution of 15.000 full-width half-maximum (from 400 to 1000 m/z). Normalized collision energy was set to 30% and one microscan was acquired for each spectrum.

Data analysis

The mass spectrometry data were searched using DIA-NN version 1.8.1 [57]. The search result was exported at the fragment ion level for integrating fractions and MaxLFQ protein quantification [58]. Differential protein expression between groups was analyzed using limma for two groups of independent samples [59]. Proteins that were detected in <25% of the samples were excluded from further analysis. Proteins with a P-value below 0.05 were selected for pathway analysis. Protein networks were acquired via the STRING database [25] and visualized in Cytoscape [60]. Protein clusters were made using ClusterONE and gene ontology (GO) analysis was carried out via the BiNGO plug-in in Cytoscape [61,62].

Metabolomics and lipidomics

Metabolomics and lipidomics were performed using snap-frozen cardiac tissue as described previously [16,17]. with some adjustments. Briefly, in a 2 mL tube containing 3 mg of freeze-dried LV cardiac tissue, the following amounts of internal standard dissolved in water were added to each sample for metabolomics: adenosine-$^{15}N_5$-monophosphate (5 nmol), adenosine-$^{15}N_5$-triphosphate (5 nmol), D_4-alanine (0.5 nmol), D_7-arginine (0.5 nmol), D_3-aspartic acid (0.5 nmol), D_3-carnitine (0.5 nmol), D_4-citric acid (0.5 nmol), $^{13}C_1$-citrulline (0.5 nmol), $^{13}C_6$-fructose-1,6-diphosphate (1 nmol), $^{13}C_2$-glycine (5 nmol), guanosine-$^{15}N_5$-monophosphate (5 nmol), guanosine-$^{15}N_5$-triphosphate (5 nmol), $^{13}C_6$-glucose (10 nmol), $^{13}C_6$-glucose-6-phosphate (1 nmol), D_3-glutamic acid (0.5 nmol), D_5-glutamine (0.5 nmol), D_5-glutathione (1 nmol), $^{13}C_6$-isoleucine (0.5 nmol), D_3-lactic acid (1 nmol), D_3-leucine (0.5 nmol), D_4-lysine (0.5 nmol), D_3-methionine (0.5 nmol), D_6-ornithine (0.5 nmol), D_5-phenylalanine (0.5 nmol), D_7-proline (0.5

nmol), $^{13}C_3$-pyruvate (0.5 nmol), D_3-serine (0.5 nmol), D_6-succinic acid (0.5 nmol), D_4-thymine (1 nmol), D_5-tryptophan (0.5 nmol), D_4-tyrosine (0.5 nmol), D_8-valine (0.5 nmol). In the same 2 mL tube, the following amounts of internal standards dissolved in 1:1 (vol/vol) methanol:chloroform were added for lipidomics: Bis(monoacylglycero)phosphate: BMP$(14:0)_2$ (0.2 nmol), Ceramide-1-phosphate: C1P (d18:1/12:0) (0.125 nmol), D_7-Cholesteryl Ester: CE(16:0) (2.5 nmol), Ceramide: Cer(d18:1/12:0) (0.125 nmol), Ceramide: Cer(d18:1/25:0) (0.125 nmol), Cardiolipin: CL$(14:0)_4$ (0.1 nmol), Diacylglycerol: DAG$(14:0)_2$ (0.5 nmol), Glucose Ceramide: GlcCer(d18:1/12:0) (0.125 nmol), Lactose Ceramide: LacCer(d18:1/12:0) (0.125 nmol), Lysophosphatidicacid: LPA(14:0) (0.1 nmol), Lysophosphatidylcholine: LPC(14:0) (0.5 nmol), Lysophosphatidylethanolamine: LPE(14:0) (0.1 nmol), Lysophosphatidylglycerol: LPG(14:0) (0.02 nmol), Phosphatidic acid: PA$(14:0)_2$ (0.5 nmol), Phosphatidylcholine: PC$(14:0)_2$ (2 nmol), Phosphatidylethanolamine: PE$(14:0)_2$ (0.5 nmol), Phosphatidylglycerol: PG$(14:0)_2$ (0.1 nmol), Phosphatidylinositol: PI$(8:0)_2$ (0.5 nmol), Phosphatidylserine: PS$(14:0)_2$ (5 nmol), Sphinganine 1-phosphate: S1P(d17:0) (0.125 nmol), Sphinganine-1-phosphate: S1P(d17:1) (0.125 nmol), Ceramide phosphocholines: SM(d18:1/12:0) (2.125 nmol), Sphingosine: SPH(d17:0) (0.125 nmol), Sphingosine: SPH(d17:1) (0.125 nmol), Triacylglycerol: TAG$(14:0)_3$ (0.5 nmol). Solvents were then added to achieve a total volume of 500 µL water, 500 µL methanol and 1 mL chloroform. Cardiac tissues were homogenized followed by addition of chloroform using a Qiagen TissueLyser II for 5 min at 30 times/s with a 5 mm Qiagen Stainless Steel Bead in each tube. All samples were mixed thoroughly and centrifuged for 10 min at 14.000 rpm.

Metabolomics

The top layer, containing the polar phase, was transferred to a clean 1.5 mL tube and dried using a vacuum concentrator at 60 °C. Dried samples were reconstituted in 100 µL 6:4 (vol/vol) methanol:water. Metabolites were analyzed using a Waters Acquity ultra-high performance liquid chromatography system coupled to a Bruker Impact II™ Ultra-High Resolution Qq-Time-Of-Flight mass spectrometer. Samples were kept at 12 °C during analysis and 5 µL of each sample was injected. Chromatographic separation was achieved using a Merck Millipore

SeQuant ZIC-cHILIC column (PEEK 100 x 2.1 mm, 3 µm particle size). Column temperature was maintained at 30 °C. Mobile phase consisted of (A) 1:9 (vol/vol) acetonitrile:water and (B) 9:1 (vol/vol) acetonitrile:water, both containing 5 mM ammonium acetate. Using a flow rate of 0.25 mL/min, the LC gradient consisted of: Dwell at 100% Solvent B, 0-2 min; Ramp to 54% Solvent B at 13.5 min; Ramp to 0% Solvent B at 13.51 min; Dwell at 0% Solvent B, 13.51-19 min; Ramp to 100% B at 19.01 min; Dwell at 100% Solvent B, 19.01-19.5 min. Column was equilibrated by increasing the flow rate to 0.4 mL/min at 100% B for 19.5-21 min. MS data were acquired using negative and positive ionization in full scan mode over the range of m/z 50-1200. Data were analyzed using Bruker TASQ software version 2.1.22.3. All reported metabolite intensities were normalized to freeze-dried tissue weight, as well as to internal standards with comparable retention times and response in the MS. Metabolite identification was based on a combination of accurate mass, (relative) retention times, ion mobility data and fragmentation spectra, compared to the analysis of a library of standards. Three samples displayed too low metabolite concentrations for reliable quantification and were excluded from further analysis.

Lipidomics

The bottom layer, containing the non-polar phase, was transferred to a clean 1.5 mL tube and evaporated under a stream of nitrogen at 60 °C. The residue was dissolved in 100 µL of 1:1 (vol/vol) methanol:chloroform. Lipids were analyzed using a Thermo Scientific Ultimate 3000 binary HPLC coupled to a Q Exactive Plus Orbitrap mass spectrometer. For normal phase separation, 2 µL of each sample was injected onto a Phenomenex® LUNA silica, 250 * 2 mm, 5 µm 100 Å. Column temperature was held at 25 °C. Mobile phase consisted of (A) 85:15 (vol/vol) methanol:water containing 0.0125% (w/vol) formic acid and 3.35 mM ammonia and (B) 97:3 (vol/vol) chloroform:methanol containing 0.0125% formic acid. Using a flow rate of 0.3 mL/min, the LC gradient consisted of: Dwell at 10% A 0-1 min, ramp to 20% A at 4 min, ramp to 85% A at 12 min, ramp to 100% A at 12.1 min, dwell at 100% A 12.1-14 min, ramp to 10% A at 14.1 min, dwell at 10% A for 14.1-15 min. For reversed phase separation, 5 µL of each sample was injected onto a Waters HSS T3 column (150 x 2.1 mm, 1.8 µm particle size).

Column temperature was held at 60 °C. Mobile phase consisted of (A) 4:6 (vol/vol) methanol:water and B 1:9 (vol/vol) methanol:isopropanol, both containing 0.1% formic acid and 10 mM ammonia. Using a flow rate of 0.4 mL/min, the LC gradient consisted of: Dwell at 100% A at 0 min, ramp to 80% A at 1 min, ramp to 0% A at 16 min, dwell at 0% A for 16-20 min, ramp to 100% A at 20.1 min, dwell at 100% A for 20.1-21 min. MS data were acquired using negative and positive ionization using continuous scanning over the range of m/z 150 to m/z 2000. Data were analyzed using an in-house developed lipidomics pipeline written in the R programming language (http://ww.r-project.org). All reported lipids were normalized to corresponding internal standards according to lipid class, as well as to freeze-dried tissue weight. Lipid identification was based on a combination of accurate mass, (relative) retention times, fragmentation spectra, analysis of samples with known metabolic defects, and the injection of relevant standards.

Quality control

Quality control was performed by repetitive injection (8 times for metabolomics; 7 times for lipidomics) of a pooled sample of all samples. Data quality was evaluated by considering the coefficient of variance in percentage for each metabolite and lipid. These data are available as a Supplemental Material ('Quality Data').

Principal component analysis

Principal component analysis of proteomics data was performed in R. Hereto, quantile normalization and log2 transformation were applied to the normalized counts. The 95th percentile was taken, the data median centered, and principal components were calculated. Principal component analyses of metabolomics and lipidomics data were performed using the R packages MixOmics version 6.18.1 and MixOmics version 6.20.0, respectively [63].

Myocardial glycogen levels

Glycogen levels were determined in cardiac tissue from NF donors, HCM myectomy samples and end-stage failing HCM hearts using a glycogen assay kit (ab65620, Abcam). In brief,

cardiac tissues (15-20 mg) were thawed on ice and quickly homogenized in an ice-cold Dounce homogenizer. Enzymes were inactivated by boiling for 10 min and supernatants were collected after centrifugation at 4 °C at 16.000 g in a microcentrifuge. Glycogen amounts were measured via enzymatic digestion and fluorometric detection, and were normalized to protein content (determined via Bradford method).

Supplemental Tables

Table S1. Clinical characteristics of NF donors and patients with obstructive HCM

	**NF Donor**	**Obstructive HCM patients**	**P value**
	(n = 20)	(n = 47)	
Sex, male	55% (11)	68% (32)	0.41
Age, years	49 ± 13	54 ± 16	0.16
BMI (kg/m^2)	27.6 ± 7.2	27.4 ± 3.7	0.60

	**HCM $G_{positive}$ (n = 19)**	**HCM $G_{negative}$ (n = 22)**	**P value**
Sex, male	68% (13)	68% (15)	>0.99
Age, years	49 ± 15	57 ± 16	0.06
BMI (kg/m^2)	26.5 ± 4.3	27.8 ± 3.4	0.30
**Dimensions**			
IVSi	11.3 ± 3.6	10.6 ± 3.3	0.40
LADi	23.6 ± 4.4	24.3 ± 3.8	0.57
**Diastolic parameters**			
E (cm/s)	83 ± 26	88 ± 34	0.63
e’ (cm/s)	5.3 ± 1.8	4.9 ± 1.4	0.45
E/e'	17.2 ± 7.6	19.0 ± 6.4	0.45
E/A	1.26 ± 0.61	1.16 ± 0.41	0.90
Deceleration time (ms)	250 ± 69	260 ± 82	0.86
**Diastolic dysfunction grade**			
1	38% (6)	26% (5)	0.72
≥2	62% (10)	74% (14)	
**Obstruction parameters**			
Rest LVOTg (mm Hg)	43.9 ± 31.0	74.1 ± 40.2	0.02*
Provoked LVOTg (mm Hg)	62.2 ± 25.6	92.1 ± 30.5	0.01*
**Medication**			
β-blocker	68% (13)	63% (14)	>0.99
Calcium channel blocker	26% (5)	32% (7)	0.74
Statins	16% (3)	23% (5)	0.70

Displayed are the mean ± standard deviation. BMI indicates body mass index; IVSi, interventricular septum thickness normalized to body surface area; LADi, left atrial diameter normalized to body surface area; LVOTg, left ventricular outflow tract gradient; $G_{negative}$, genotype-negative and $G_{positive}$, genotype-positive. * P<0.05

Table S2 is available as a separate supplemental material file.

Table S3. Overview of lipid names and abbreviations

**Abbreviation**	**Full name**	**Lipid category**
TG	Triacylglycerol	Triradylglycerols
TG[O]	Alkyldiacylglycerol	Triradylglycerols
DG	Diacylglycerol	Diradylglycerols
DG[O]	Alkylacylglycerol	Diradylglycerols
DG[P]	Alkenylacylglycerol	Diradylglycerols
MG	Monoacylglycerol	Monoradylglycerols
MG[O]	Alkylglycerol	Monoradylglycerols
AC	Acylcarnitine	Fatty esters
AC-OH	Hydroxyacylcarnitine	Fatty esters
FA	Fatty acids	Fatty acids and conjugates
FA-OH	Hydroxy fatty acids	Fatty acids and conjugates
C1P[d]	Ceramide-1-phosphate	Ceramides
Cer[d]	Ceramide	Ceramides
SM[d]	Sphingomyelin	Phosphosphingolipids
SM[t]	Hydroxysphingomyelin	Phosphosphingolipids
SPH[d]	Sphingosine/Sphinganine	Sphingoid bases
Hex2Cer[d]	Dihexosylceramide	Neutral glycosphingolipids
Hex2Cer[t]	Hydroxy-dihexosylceramide	Neutral glycosphingolipids
Hex3Cer[d]	Trihexosylceramide	Neutral glycosphingolipids
Hex3Cer[t]	Hydroxy-trihexosylceramide	Neutral glycosphingolipids
HexCer[d]	Hexocylceramide	Neutral glycosphingolipids
HexCer[t]	Hydroxy-hexosylceramide	Neutral glycosphingolipids
CE	Cholesteryl ester	Sterols
PC	Phosphatidylcholine	Glycerophosphocholines
PC[O]	Alkylphosphatidylcholine	Glycerophosphocholines
PC[P]	Alkenylphosphatidylcholine	Glycerophosphocholines
1-acyl LPC	2-Lysophosphatidylcholine	Glycerophosphocholines
2-acyl LPC	1-Lysophosphatidylcholine	Glycerophosphocholines
LPC	Lysophosphatidylcholine	Glycerophosphocholines
LPC[O]	Alkyllysophosphatidylcholine	Glycerophosphocholines
LPC[P]	Alkenyllysophosphatidylcholine	Glycerophosphocholines
PE	Phosphatidylethanolamine	Glycerophosphoethanolamines
PE[O]	Alkylphosphatidylethanolamine	Glycerophosphoethanolamines
PE[P]	Alkenylphosphatidylethanolamine	Glycerophosphoethanolamines
1-acyl LPE	2-Lysophosphatidylethanolamine	Glycerophosphoethanolamines
2-acyl LPE	1-Lysophosphatidylethanolamine	Glycerophosphoethanolamines
LPE	Lysophosphatidylethanolamine	Glycerophosphoethanolamines
LPE[O]	Alkyllysophosphatidylethanolamine	Glycerophosphoethanolamines
LPE[P]	Alkenyllysophosphatidylethanolamine	Glycerophosphoethanolamines
PA	Phosphatidic acid	Glycerophosphates
LPA	Lysophosphatic acid	Glycerophosphates
PG	Phosphatidylglycerol	Glycerophosphoglycerols

LPG	Lysophosphatidylglycerol	Glycerophosphoglycerols
PI	Phosphatidylinositol	Glycerophosphoinositols
LPI	Lysophosphatidylinositol	Glycerophosphoinositols
PS	Phosphatidylserine	Glycerophosphoserines
LPS	Lysophosphatidylserine	Glycerophosphoserines
CL	Cardiolipin	Glycerophosphoglycerophosphoglycerols
DLCL	Dilysocardiolipin	Glycerophosphoglycerophosphoglycerols
MLCL	Monolysocardiolipin	Glycerophosphoglycerophosphoglycerols
BMP	Lysobisphosphatidic acid	Glycerophosphoglycerols
SLBPA	Semi-lysobisphosphatidic acid	Glycerophosphoglycerols
GA1[d]	GA1-ganglioside	Gangliosides
GA1[t]	GA1-hydroxyganglioside	Gangliosides
GM3[d]	GM3-ganglioside	Gangliosides

Table S4. Sample sizes and demographics of non-failing (NF) donors and patients with hypertrophic cardiomyopathy (HCM) in previous studies into the lipidome and/or metabolome in cardiac tissue of patients with HCM

Study	Sample size metabolomics/lipidomics		Genotype positive (%)	Age		Sex (% male)		BMI	
	NF Donor	HCM		NF Donor	HCM	NF Donor	HCM	NF Donor	HCM
Ranjbarvaziri	6	13	62	47 ± 17	54 ± 13	33	69	Not reported	Not reported
Previs*	8	28	71	52 ± 6	45 ± 13#	38	46#	Not reported	33 ± 8#
Wang	16	349	58	39 ± 18	45 ± 15	85	60	Not reported	25 ± 4
Nollet	12	47	40	54 ± 8	54 ± 16	50	68	28 ± 7	27 ± 4

*Only metabolomics was performed

Based on entire study population. Demographics of subset of samples that underwent metabolomics and lipidomics not reported

Values represent mean ± standard deviation. BMI indicates body mass index.

Table S5 is available as a separate supplemental material file.

Supplemental Figures

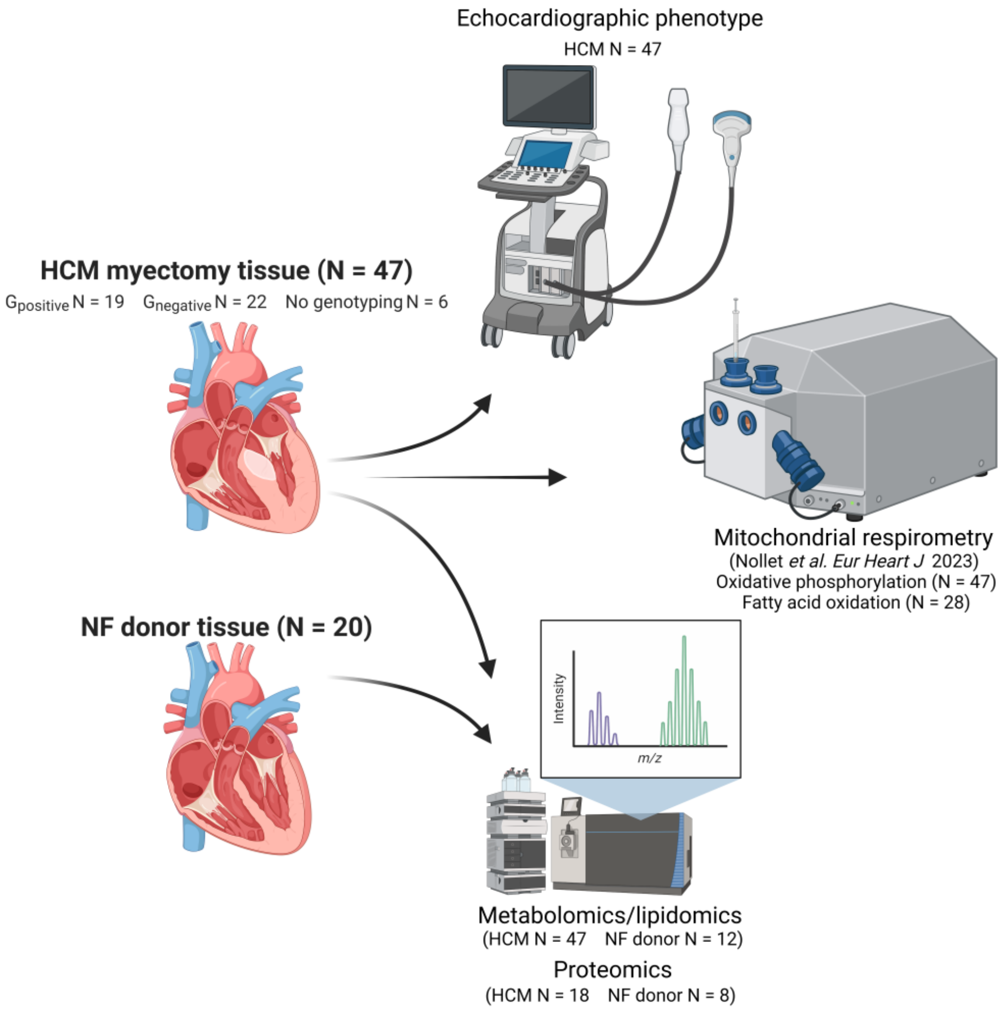

Figure S1. Study design. A total of 47 myectomy tissue samples from patients with obstructive hypertrophic cardiomyopathy (HCM) and 20 non-failing (NF) donor tissue samples were used. Next-generation sequencing revealed presence of a causative mutation in 19 patients (genotype-positive; $G_{positive}$); 24 patients were found to be genotype-negative ($G_{negative}$); 6 patients were not tested. Metabolomics and lipidomics were performed on 47 HCM samples and 12 NF donor samples; 18 HCM samples and 8 NF donor samples were analyzed via proteomics. Previously generated mitochondrial respiration data [15] was analyzed in this study in relation to metabolomic and lipidomic data. Created with BioRender.com

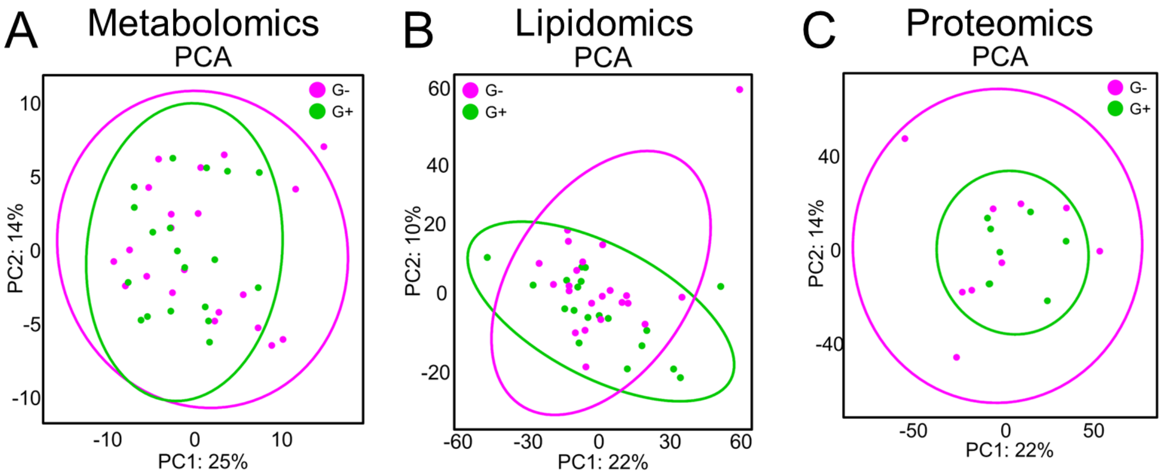

Figure S2. No separate clustering of genotype-positive (G+; $G_{positive}$) and genotype-negative (G-; $G_{negative}$) patients in terms of metabolomic, lipidomic and proteomic profile. Principal component analysis (PCA) showing no distinct clustering of $G_{positive}$ and $G_{negative}$ patients with HCM at the level of metabolites **(A)**, lipids **(B)** and proteins **(C)**.

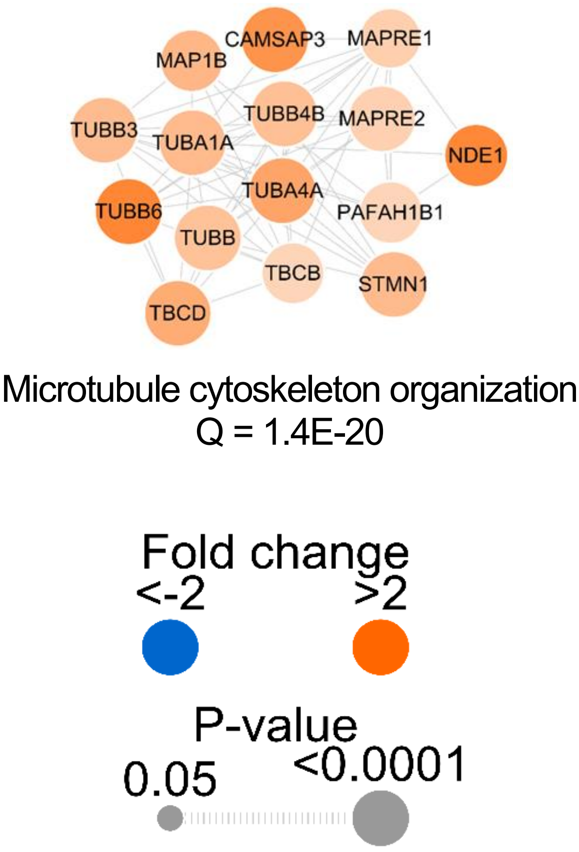

Figure S3. Upregulation of microtubule cytoskeleton-related proteins in cardiac tissue from patients with hypertrophic cardiomyopathy (HCM) vs non-failing (NF) donor tissue. Color and size of protein nodes indicate fold change between groups and significance of these changes, respectively.

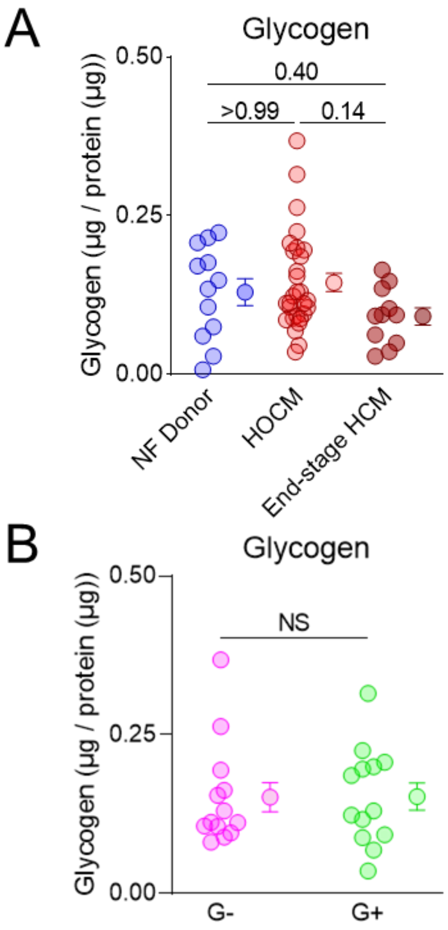

Figure S4. No differences in glycogen levels in hypertrophic cardiomyopathy (HCM) hearts vs non-failing (NF) donors at any disease stage. (A) shows levels of glycogen in cardiac tissue from NF donors, obstructive HCM (HOCM) and end stage HCM. **(B)** shows levels of glycogen in cardiac tissue from genotype-negative ($G_{negative}$; G-) and genotype-positive ($G_{positive}$; G+) patients with obstructive HCM. **(A)** was analyzed via 1-way ANOVA; **(B)** was analyzed via Mann-Whitney test.

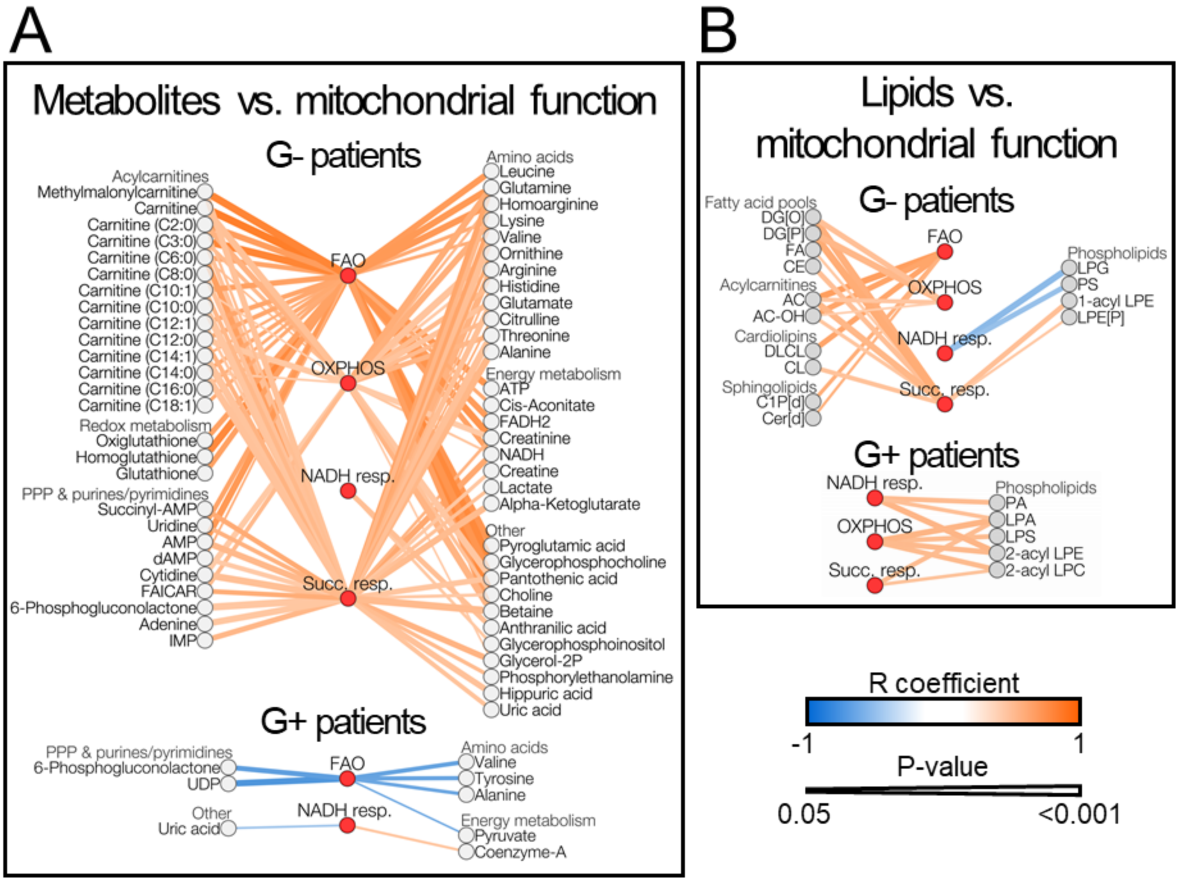

Figure S5. Correlations between mitochondrial respiration parameters and metabolites and lipids in patients with hypertrophic cardiomyopathy (HCM). Correlations were tested via linear regression and are described by Pearson's R coefficient. **(A)** shows all significant correlations between mitochondrial respiration parameters and metabolites in genotype-negative ($G_{negative}$; G-) and genotype-positive ($G_{positive}$; G+) patients; **(B)** shows all significant correlations between mitochondrial respiration parameters and lipids classes in $G_{negative}$ and $G_{positive}$ patients. Blue and orange lines indicate negative and positive correlations, respectively. Line thickness corresponds with significance level of the correlation. FAO indicates fatty acid oxidation; OXPHOS, oxidative phosphorylation capacity; NADH resp., NADH-linked respiration; Succ. resp., succinate-linked respiration. Exact R coefficients and P-values, as well as group comparison statistics between non-failing donors and G- or G+ patients of metabolites and lipids in these correlations are available as a Supplemental Material ('Correlation statistics').

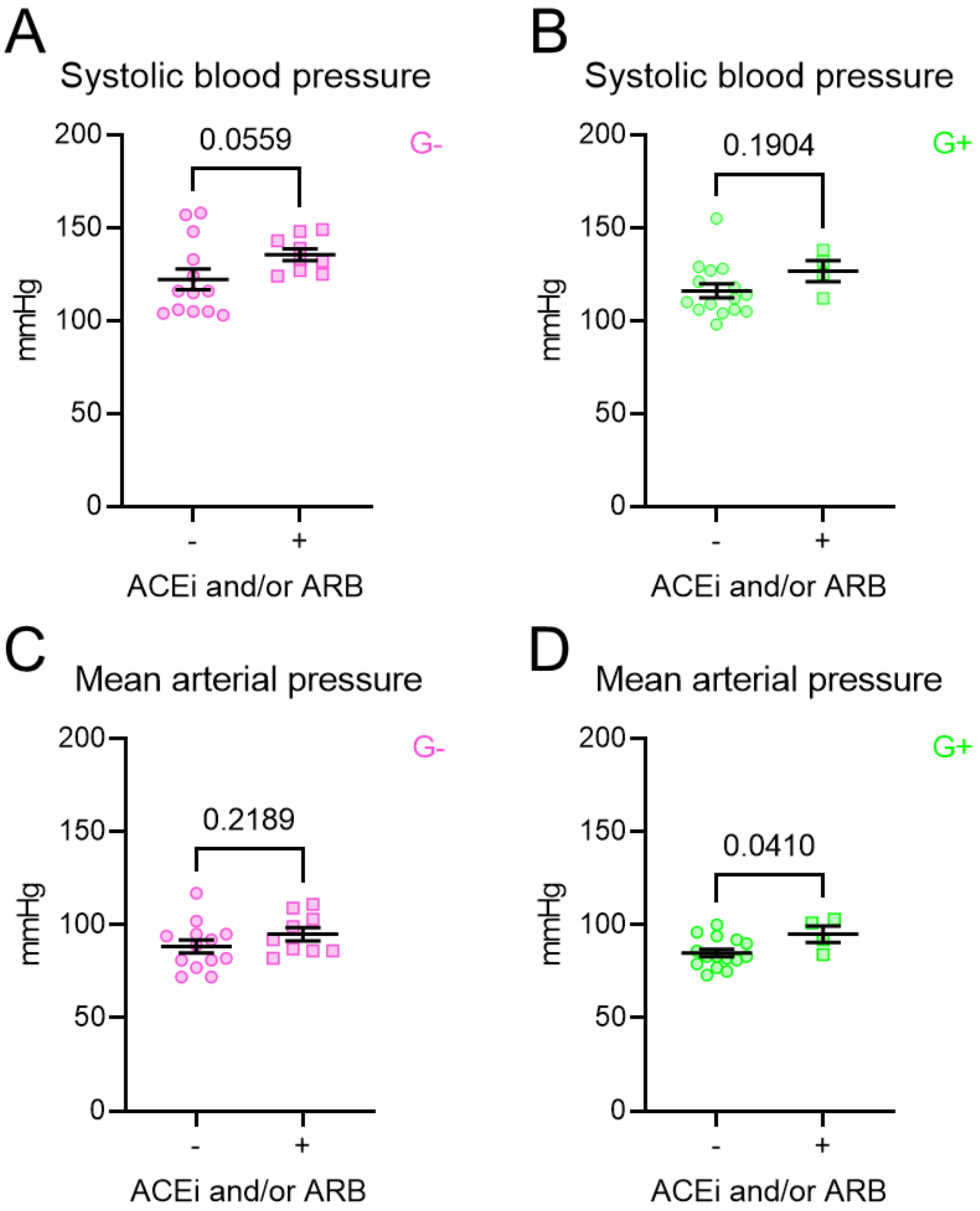

Figure S6. Systolic blood pressure and mean arterial pressure in genotype-negative (G-) and genotype-positive (G+) patients with hypertrophic cardiomyopathy on and off angiotensin converting enzyme inhibitors (ACEi) and/or angiotensin receptor blockers (ARB). (A) was analyzed via Welch's t-test; **(B, C, D)** were analyzed via student's t-test.

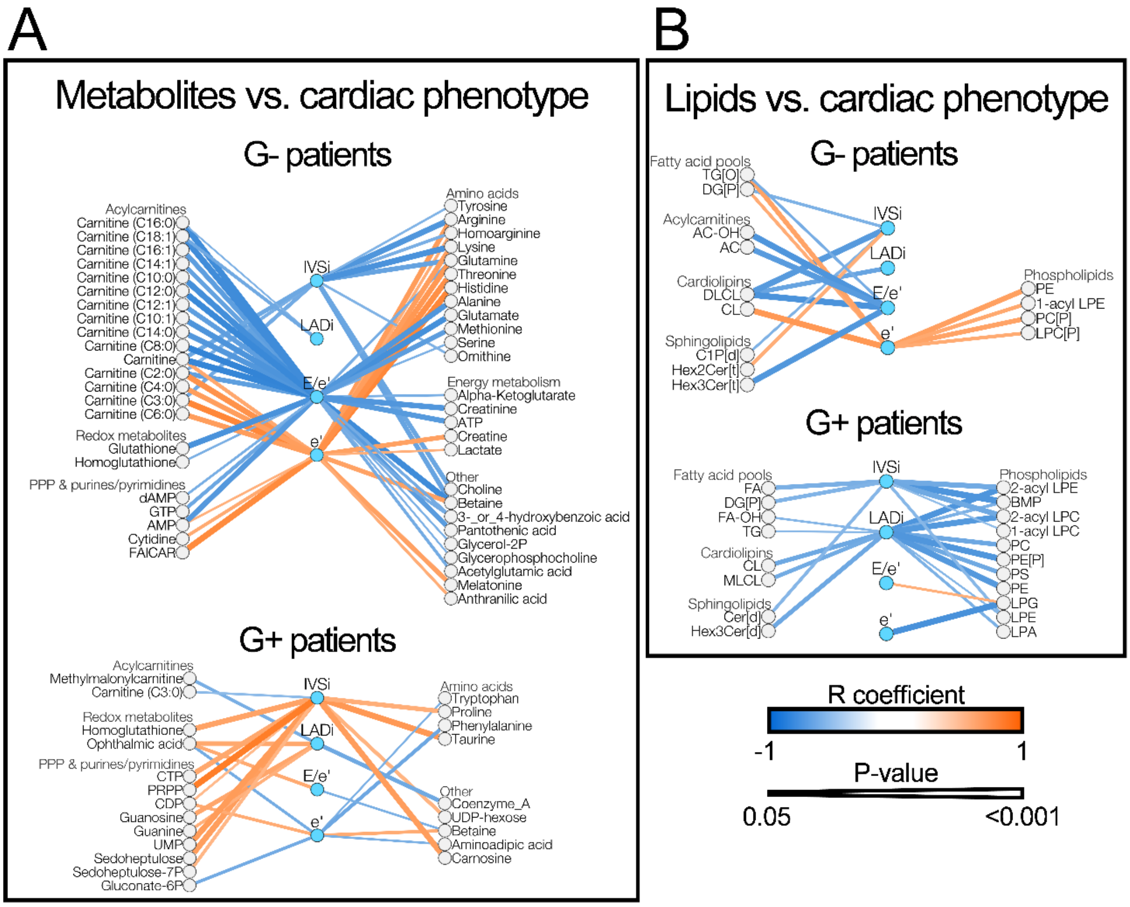

Figure S7. Correlations between cardiac phenotype and metabolites and lipids in patients with hypertrophic cardiomyopathy (HCM) not receiving angiotensin-converting enzyme inhibitors or angiotensin receptor blockers. Correlations were tested via linear regression and are described by Pearson's R coefficient. **(A)** shows all significant correlations between cardiac parameters and metabolites in genotype-negative ($G_{negative}$; G-) and genotype-positive ($G_{positive}$; G+) patients; **(B)** shows all significant correlations between cardiac parameters and lipids classes in $G_{negative}$ and $G_{positive}$ patients. Blue and orange lines indicate negative and positive correlations, respectively. Line thickness corresponds with significance level of the correlation. IVSi indicates septal thickness indexed to body surface area; LADi, left atrial diameter indexed to body surface area. Exact R coefficients and P-values, as well as group comparison statistics between non-failing donors and G- or G+ patients of metabolites

and lipids in these correlations are available as a Supplemental Material ('Correlation statistics').
